# Effect of the Family-Centered Advance Care Planning for Teens with Cancer Intervention on Sustainability of Congruence About End-of-Life Treatment Preferences: A Randomized Clinical Trial

**DOI:** 10.1001/jamanetworkopen.2022.20696

**Published:** 2022-07-12

**Authors:** Jennifer Susan Needle, Sarah Friebert, Jessica D. Thompkins, Daniel H. Grossoehme, Justin N. Baker, JiJi Jiang, Jichuan Wang, Maureen E. Lyon

**Affiliations:** 1 Department of Pediatrics and Center for Bioethics, University of Minnesota, Minneapolis; 2 Haslinger Family Pediatric Palliative Care Center, Akron Children’s Hospital, Akron, Ohio; 3 Rebecca D. Considine Research Institute, Akron Children’s Hospital, Akron, Ohio; 4 Division of Allergy and Immunology, Children’s National Hospital, Washington, DC; 5 Division of Quality of Life and Palliative Care, St Jude Children’s Research Hospital, Memphis, Tennessee; 6 Henry M Jackson Foundation for the Advancement of Military Medicine, Bethesda, Maryland; 7 Division of Biostatistics and Study Methodology, Center for Translational Research/Children’s National Research Institute, Children’s National Hospital, Washington, DC; 8 Center for Translational Research/Children’s National Research Institute, Children’s National Hospital, Washington, DC; 9 Department of Pediatrics, George Washington University School of Medicine and Health Sciences, Washington, DC; 10 Division of Adolescent and Young Adult Medicine, Children’s National Hospital, Washington, DC

## Abstract

**Importance:**

The effect of pediatric advance care planning (pACP) on the sustainability of end-of-life treatment preference congruence between adolescents with cancer and their families has not been examined.

**Objective:**

To evaluate the longitudinal efficacy of the Family-Centered Advance Care Planning for Teens with Cancer (FACE-TC) intervention to sustain adolescent-family congruence about end-of-life treatment preferences.

**Design, Setting, and Participants:**

This multisite, assessor-blinded, randomized clinical trial enrolled adolescents with cancer (aged 14-21 years) and their family members from 4 pediatric hospitals between July 16, 2016, and April 30, 2019. Participants were randomized 2:1 to FACE-TC (intervention group) or treatment as usual (control group) and underwent 5 follow-up visits over an 18-month postintervention period. Intention-to-treat analyses were conducted from March 9, 2021, to April 14, 2022.

**Exposures:**

Adolescent-family dyads randomized to the FACE-TC group received 3 weekly 60-minute sessions consisting of the discussion and/or completion of the Lyon Family-Centered Advance Care Planning Survey (session 1), Respecting Choices Next Steps pACP conversation (session 2), and Five Wishes advance directive (session 3). Dyads in the control group received treatment as usual. Both groups received pACP information.

**Main Outcomes and Measures:**

Congruence was measured by completion of the Statement of Treatment Preferences (a document that discusses 4 hypothetical clinical situations and treatment choices for each scenario: continue all treatments, stop all efforts to keep me alive, or unsure) after session 2 (time 1) and at 3 months (time 2), 6 months (time 3), 12 months (time 4), and 18 months (time 5) after intervention. The influence of FACE-TC on the trajectory of congruence over time was measured by longitudinal latent class analysis.

**Results:**

A total of 252 participants (126 adolescent-family dyads) were randomized. Adolescents (mean [SD] age, 17 [1.9] years) and family members (mean [SD] age, 46 [8.3] years) were predominantly female (72 [57%] and 104 [83%]) and White individuals (100 [79%] and 103 [82%]). There was an 83% (104 of 126) retention at the 18-month assessment. Two latent classes of congruence over time were identified: high-congruence latent class (69 of 116 [60%]) and low-congruence latent class (47 of 116 [41%]). The dyads in the FACE-TC group had a 3-fold odds of being in the high-congruence latent class (odds ratio [OR], 3.22; 95% CI, 1.09-9.57) compared with the control group. Statistically significant differences existed at 12 months (β [SE] = 1.17 [0.55]; *P* = .03]) but not at 18 months (OR, 2.08; 95% CI, 0.92-4.69). In the high-congruence latent class, good agreement (agreement on 2 or 3 of 4 situations) increased over 12 months. White adolescents and families had significantly greater odds of congruence than a small population of American Indian or Alaska Native, Asian, Black or African American, Hispanic or Latino, or multiracial adolescents and families (OR, 3.97; 95% CI, 1.07-14.69).

**Conclusions and Relevance:**

Results of this trial showed that, for those who received the FACE-TC intervention, the families’ knowledge of their adolescents’ end-of-life treatment preferences was sustained for 1 year, suggesting yearly follow-up sessions. Race and ethnicity–based differences in the sustainability of this knowledge reflect a difference in the effect of the intervention and require further study.

**Trial Registration:**

ClinicalTrials.gov Identifier: NCT02693665

## Key Points

**Question:**

Does the Family-Centered Advance Care Planning for Teens with Cancer (FACE-TC) intervention sustain agreement about end-of-life treatment preferences 18 months after the intervention?

**Findings:**

In this randomized clinical trial involving 126 adolescent-family dyads, those who received FACE-TC had a 3-fold greater odds of being in the high-congruence class over 12 months, but this significant difference was not sustained at 18 months. Adolescents in the FACE-TC group were significantly more likely to have an advance directive in the electronic health record at study closeout than those in the treatment as usual group (80% vs 19%).

**Meaning:**

Findings of this trial suggest that FACE-TC participation improved congruence between adolescents and their families regarding end-of-life treatment preferences for 1 year; yearly follow-up sessions are indicated.

## Teaser

This randomized clinical trial examines an intervention that supports and enables the discussion of advance directives between adolescents with cancer and their families.

## Introduction

Adolescents with a serious illness, their families, and clinicians concur that pediatric advance care planning (pACP) is appropriate.^1,2,3,4^ Key elements of pACP in this population^1,2,3,4,5,6^ include (1) giving adolescents a voice in their own end-of-life care, (2) initiating early pACP conversations to close gaps in understanding,^7^ (3) documenting advance directives in the electronic health record (EHR), and (4) sharing pACP conversations with the treating clinicians to support communication before a medical crisis.^8^ Various models of pACP for adolescents with a serious illness are being developed,^9,10,11,12,13^ along with tools to guide conversations.^14,15,16^

Although ongoing conversations are recommended,^17,18^ optimal timing has not been empirically demonstrated.^19,20^ Timing is important for adolescents with cancer, the leading cause of disease-related death in this age group.^21,22,23^ Adolescents with cancer prefer to talk about pACP from the time of diagnosis and not while hospitalized or if they are dying.^7,24^ Determining whether early pACP conversations are effective over time is critical to patient-centered or family-supported care. The unanswered question is whether families can sustain knowledge of the patients’ treatment preferences over time, even as these preferences change, as demonstrated among adolescents^25^ and adults^26^ with HIV.

A theoretically guided, single-site, 3-session intervention called Family-Centered Advance Care Planning for Teens with Cancer (FACE-TC) was pilot-tested and informed by community-based participatory research.^27^ The pilot trial resulted in congruence about end-of-life treatment preferences.^28,29^ The FACE (Family-Centered) model has also been pilot-tested in adolescents and young adults, aged 14 to 27 years, who underwent bone marrow transplant.^30^ In that study, the participants were capable of meaningful deliberation about future treatment choices,^30^ and their decision-making was influenced by consideration for family, quality of life, and awareness of self.^31^

The primary aim of this randomized clinical trial was to evaluate the longitudinal efficacy of FACE-TC to sustain adolescent-family congruence about end-of-life treatment preferences. We hypothesized that (1) participants who received FACE-TC would be better at retaining congruence over time compared with participants in the control group who received treatment as usual, (2) the development of congruence would not be homogeneous and that FACE-TC would influence congruence trajectories over time, and (3) adolescents who received FACE-TC would be more likely to have advance directives documented in the EHR at the study closeout compared with control adolescents.

## Methods

The institutional review board at each study site approved the trial protocol (Supplement 1), and an external safety monitoring committee monitored the trial. Participants provided written informed consent or assent. We followed the Consolidated Standards of Reporting Trials (CONSORT) reporting guideline.

### Study Design, Participants, and Setting

Full details of the trial have been published.^32^ Briefly, this multisite, assessor-blinded, intention-to-treat randomized clinical trial enrolled adolescent-family dyads between July 16, 2016, and April 30, 2019. Dyads were recruited from 4 quaternary pediatric hospitals: Akron Children’s Hospital (Akron, Ohio), Children’s National Hospital (Washington, DC), St Jude Children’s Research Hospital (Memphis, Tennessee), and University of Minnesota Masonic Children’s Hospital (Minneapolis, Minnesota).

Inclusion criteria for adolescents were diagnosis of cancer, 14 to 21 years of age at enrollment, no intellectual disabilities that would interfere with decision-making, ability to speak English (see protocol for Spanish speakers^13^), and not in foster care. Eligibility criteria for a family member were as a legal guardian (if the adolescent was younger than 18 years) or as the chosen surrogate decision maker (if the adolescent was 18 years or older at enrollment), ability to cognitively engage, ability to speak English, and knowledge of the adolescent’s diagnosis. After enrolling, all participants underwent secondary screening for the exclusion criteria: severe depression,^33^ homicidality,^34^ suicidality,^33^ and/or psychosis.^34^

Participants received compensation in the amount of $25 for visits 1 to 4 and $35 for visits 5 to 8. Race and ethnicity were identified by participants, per study funder requirements.

### Procedures and Randomization

We completed training in the trial protocol. We conducted yearly booster sessions for study staff. Interventionists (including J.D.T.) were nurses who received certification in the Respecting Choices Next Steps program, which required online education about advance care planning (ACP) with continuing education credits and 12 hours of face-to-face training.^35,36^ One of us (M.E.L.) provided monthly group supervision to interventionists by conference call, which involved video review of the Respecting Choices session and competency criteria checklist.

After consulting with adolescents’ primary oncologists, assessors approached potentially eligible participants face-to-face during outpatient appointments. Visit 1 included enrollment; secondary screening; and, if eligible, completion of baseline questionnaires.

**Figure 1.**
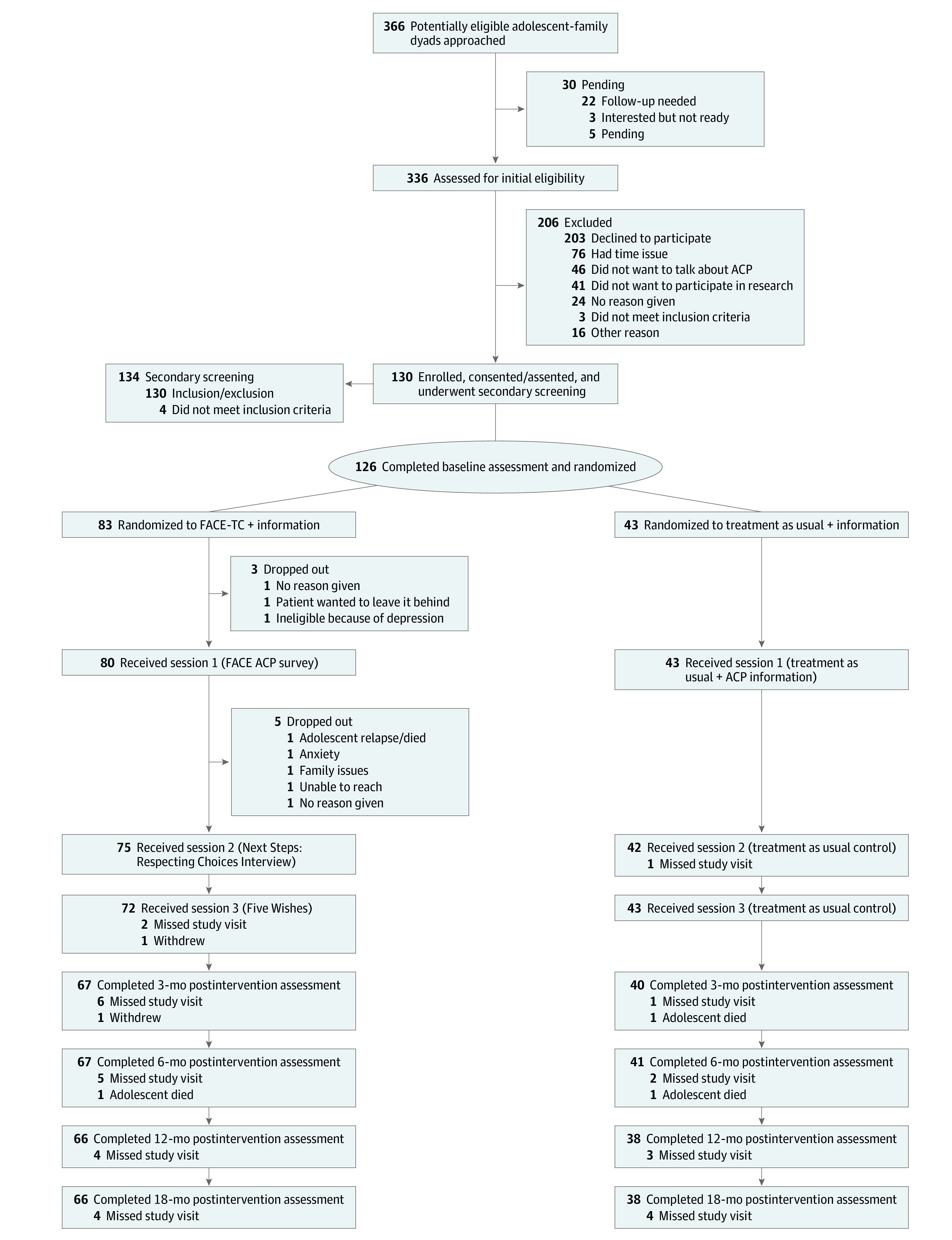
Study Flow Diagram of the Family-Centered Advance Care Planning for Teens with Cancer (FACE-TC) Trial Using Intention-to-Treat Design ACP indicates advance care planning; FACE, Family-Centered.

Dyads were randomized 2:1 to either the FACE-TC intervention or treatment as usual (Figure 1). Completion of baseline assessments in the REDCap (Research Electronic Data Capture; Vanderbilt University) database triggered randomization using a computer-generated randomization table. Interventionists notified the participants of their group assignment to protect assessor blinding. Blinded assessors read study questionnaires aloud to the participants. Questionnaires were administered separately to adolescents and their families.

### Exposures

All dyads received usual care and a pACP booklet.^37^ Dyads who were randomized to the control group received treatment as usual. Dyads who were randomized to FACE-TC received three 60-minute sessions that were conducted weekly. In session 1, the Lyon Family-Centered Advance Care Planning Survey^7^ was administered separately to the adolescent and family member to prepare them for issues to be discussed in the next 2 sessions. In session 2, the Respecting Choices Next Steps pACP conversation guide^35,36^ was discussed with the dyad, with the adolescent taking the lead, to explore the adolescent’s understanding of their cancer and its potential complications, experiences with death and dying, and end-of-life treatment preferences. In session 3, the Five Wishes,^38^ a legal advance directive in most US states, was completed by both the adolescent and family member. For adolescents younger than 18 years, their legal guardian’s signature was required on the Five Wishes document. A copy was given to the family member.

The results and documents from these FACE-TC sessions were then communicated to clinicians in an email by the interventionist, who also entered the documents into the EHR. eTable 1 in Supplement 2 describes the goals and processes of each session.

### Primary Outcome Measure 

The Statement of Treatment Preferences is a structured and standardized way to document adolescents’ specific treatment preferences. Family members completed a Statement of Treatment Preferences form separately according to their understanding of the adolescent’s preferences. Four cancer-specific situations and the benefits and burdens of treatment options were discussed: (1) long hospitalization with low chance of survival; (2) treatment to extend life by no more than 3 months, with serious adverse effects; (3) physical impairment; and (4) profound cognitive impairment. Choices for each situation were as follows: to continue all treatments so I could live as long as possible (staying alive is most important to me no matter what), to stop all efforts to keep me alive (my definition of living well is more important than length of life), or unsure. The Statement of Treatment Preferences method has been used in multiple adolescent^25,28,30^ and adult studies,^26,39^ increasing its replicability.

Dyadic responses for data analysis were recoded into 2 categories for each situation: congruent vs noncongruent. Unsure responses were coded as noncongruent (ie, no agreement to take a course of action). As a data-reduction strategy, 3 levels of overall agreement were generated based on preferences on the 4 cancer-related situations (poor congruence, agreement in 0-1 situation; good congruence, agreement in 2-3 situations; perfect congruence, agreement in all 4 situations).

### Secondary and Exploratory Outcomes Measures

We developed a standardized EHR-based form for data abstraction of any goals of care or advance directives in the EHR before the study and at study closeout.^29^ Goals-of-care conversations were operationalized as a progress note, family meeting note, or palliative care note in the EHR concerning illness understanding or end-of-life treatment preferences. Data abstraction was conducted by a blinded assessor.

### Demographic Characteristics

A standardized form was used to collect self-reported age, sex, gender, and race and ethnicity as well as family-reported educational level and employment status. Reported household income and household size determined the 2016 federal poverty level of the participants. Data abstraction included cancer diagnosis, date of diagnosis, history of relapse, history of bone marrow transplant, and on or off active treatment status.

### Statistical Analysis

All intention-to-treat analyses were by original randomization. We used longitudinal latent class analysis (LLCA)^40,41,42,43,44,45^ to explore potential latent classes of dyads with respect to trajectories of congruence over time. Longitudinal latent class analysis characterizes both within-person variation and between-person variation and identifies latent classes according to groupings of similar patterns of outcome growth trajectories across time with no assumption about the form of outcome change (ie, an agnostic, data-driven approach). The LLCA models with 3 time points were estimated as time 1 to time 3, time 1 to time 4, and time 1 to time 5. Description of the congruence measure by latent classes is provided in the eAppendix 2 in Supplement 2.

Next, we built a logistic regression model using a 3-step method to estimate the effect of FACE-TC, while controlling for covariates. Details on the LLCA are provided in the eAppendix 1 in Supplement 2. All models were estimated using Mplus, version 8.6 (Muthén and Muthén).^45^

To evaluate the efficacy of FACE-TC for early completion of pACP goals of care and advance directives, we used χ^2^ statistics to assess the difference in completion of pACP goals of care and advance directives between participants in the FACE-TC group and those in the control group. Next, exact logistic regression was applied to test the effect of FACE-TC on documentation of goals of care and advance directives in the EHR, while controlling for sociodemographic characteristics.

Data were entered into REDCap, version 8.10.18-2020.^46^ Statistical significance was set at α = .05. Analyses were conducted from March 9, 2021, to April 14, 2022, using SAS, version 9.4 (SAS Institute Inc).

## Results

### Participant Characteristics

A total of 252 participants (126 adolescent-family dyads) were randomized (Figure 1). Adolescents had a mean (SD) age of 17 (1.9) years; 72 participants were female (57%), 54 were male (43%), and 100 were White individuals (79%) (Table 1). Family members had a mean (SD) age of 46 (8.3) years; were predominantly female (104 [83%]) and White (103 [82%]) individuals; and included 94 mothers (75%), 19 fathers (15%), and 10 adolescent-chosen nonbiological surrogate decision makers (8%). There were no study-related adverse events.

**Table 1.** Baseline Demographic Characteristics of Adolescents With Cancer and Their Families

Characteristic	Adolescents, No. (%)		Families, No. (%)	
	FACE-TC group	Treatment as usual group	FACE-TC group	Treatment as usual group
No. of participants	83	43	83	43
Age, mean (SD) [range], y	16.9 (1.8) [14-20]	17.0 (2.0) [14-20]	45.6 (8.2) [19-67]	46.5 (8.4) [20-63]
Sex				
Female	45 (54)	27 (63)	67 (81)	37 (86)
Male	38 (46)	16 (37)	16 (19)	6 (14)
Race^a^				
American Indian or Alaska Native	0	0	0	1 (2)
Asian	3 (4)	0	3 (4)	0
Black or African American	12 (14)	5 (12)	10 (12)	4 (9)
White	63 (76)	37 (86)	68 (82)	35 (81)
More than 1 race	4 (5)	1 (2)	2 (2)	3 (7)
Declined to answer	1 (1)	0	0	0
Ethnicity^a^				
Hispanic or Latino	5 (6)	0	4 (5)	0
Not Hispanic or Latino	76 (92)	40 (93)	79 (95)	42 (98)
Declined to answer	2 (2)	3 (7)	0	1 (2)
Educational level				
No high school diploma or GED equivalency	45 (54)	26 (60)	2 (2)	0
High school or GED equivalency	28 (34)	8 (19)	16 (19)	7 (16)
Some college but no bachelor’s degree	9 (11)	9 (21)	31 (37)	17 (40)
Bachelor’s, master’s, doctoral, or professional degree	0	0	34 (41)	19 (44)
Declined to answer	1 (1)	0	0	0
Household income, FPL				
Equal to or below	NA	NA	21 (25)	12 (28)
101%-200%	NA	NA	23 (28)	14 (33)
201%-300%	NA	NA	14 (17)	5 (12)
>300%	NA	NA	23 (28)	10 (23)
Declined to answer	NA	NA	2 (2)	2 (5)
Relationship				
Biological	NA	NA	76 (92)	37 (86)
Nonbiological	NA	NA	7 (8)	6 (14)
On active treatment?				
Yes	20 (24)	7 (16)	NA	NA
No	63 (76)	36 (84)	NA	NA
Cancer diagnosis				
Leukemia	26 (31)	16 (37)	NA	NA
Solid tumors	21 (25)	8 (19)	NA	NA
Brain tumor	16 (19)	8 (19)	NA	NA
Lymphoma	10 (12)	9 (21)	NA	NA
Other^b^	10 (12)	2 (5)	NA	NA
Length of time since diagnosis, mean (SD) [range], mo	81 (68) [1-232]	70 (60) [2-198]	NA	NA

Abbreviations: FACE-TC, Family-Centered Advance Care Planning for Teens with Cancer; FPL, federal poverty level; GED, General Educational Development; NA, not applicable.

^a^ Race and ethnicity were self-reported by participants.

^b^ Other included totally resected World Health Organization grade II ependymoma, Ewing sarcoma in left scalp, germ cell tumor of right testis, nonosseous retroperitoneal Ewing sarcoma, papillary thyroid carcinoma, plexiform neurofibroma, Wilm tumor in left kidney, melanoma, and GATA2-related myelodysplastic syndrome, neuroblastoma, pilonidal abscess, synovial sarcoma of soft tissue, and metastatic melanoma.

The primary reason for declining participation was lack of time (76 of 203 dyads [37%]), whereas 46 dyads (23%) declined because at least 1 member of the dyad did not want to discuss ACP (Figure 1). Male adolescents were significantly more likely to decline participation (difference of 14%; 95% CI, 4%-25%; *P* = .02). There were no significant between-group differences in age, race and ethnicity, cancer diagnosis, and active treatment status between enrollees and decliners.

Retention was 83% (104 of 126 dyads) at 18 months. Benchmarks were achieved: (1) enrollment of 130 dyads, (2) retention of more than 70% of dyads at the 18-month assessment, and (3) 90% of participants who began session 1 completed session 3 of the FACE-TC intervention.

**Figure 2.**
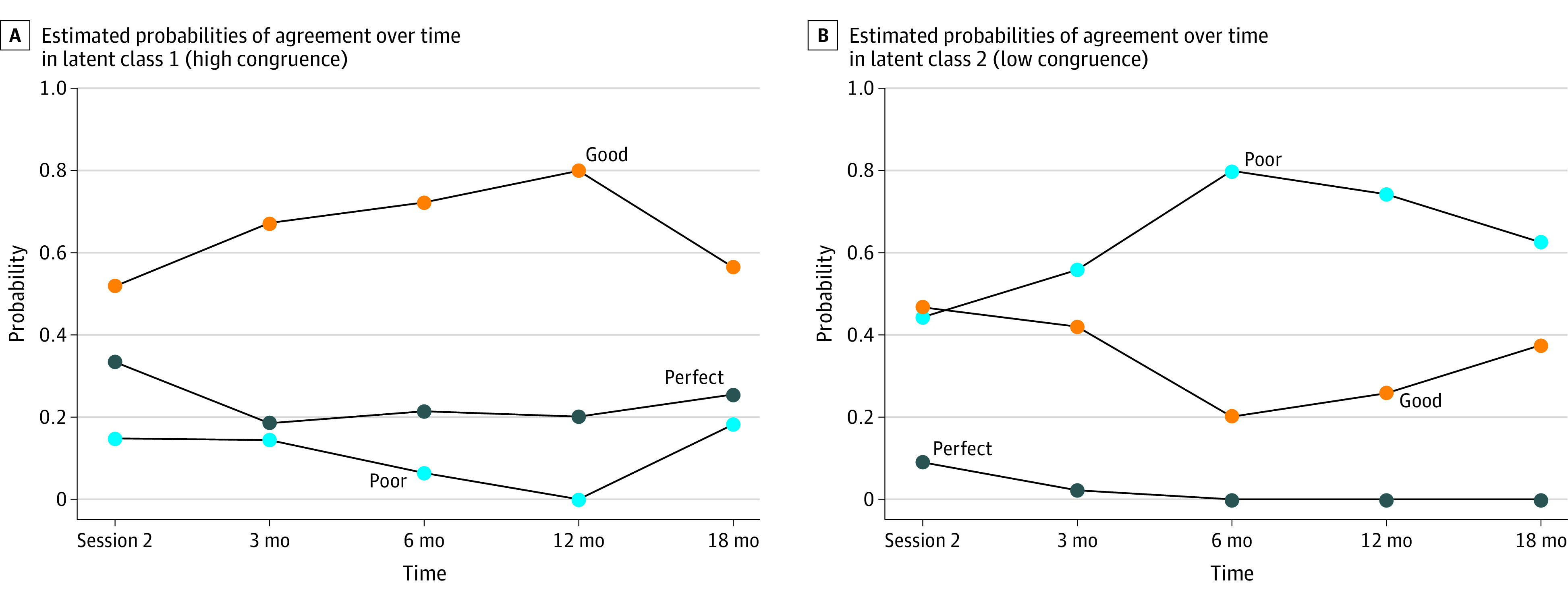
Probability of Congruence Change Trajectories Longitudinal latent class models were used to estimate the probability of congruence over time for each adolescent-family dyad and to classify dyads into groups with similar patterns. Lines indicate levels of agreement (poor, good, or perfect).

The LLCA models were estimated with different time points immediately after session 2 for dyads in the FACE-TC group or 3 weeks after baseline for the treatment as usual group (time 1) and at 3 months (time 2), 6 months (time 3), 12 months (time 4), and 18 months (time 5) after intervention. Two distinct latent classes were identified using 12 months of data: high-congruence latent class (69 of 116 [60%]) and low-congruence latent class (47 of 116 [41%]). Selected results of the 4 time data (time 1-time 4) of the LLCA for levels of agreement over time by latent class are shown in Figure 2 and eTable 2 in Supplement 2. The probabilities of poor agreement were lower in the high-congruence latent class and reached 0 at time 4 (Figure 2A) compared with the low-congruence latent class (Figure 2B). In the high-congruence latent class, FACE-TC affected the trajectory of congruence, with good agreement (agreement on 2 or 3 of 4 situations) increasing over 12 months and perfect agreement decreasing from time 1 to time 2 and then stabilizing (Figure 2A). Overall, dyadic agreement levels (poor, good, and perfect) varied across latent classes at each time point.

Table 2 shows the actual effect of FACE-TC on class membership, with the mean probability of congruence. The dyads in the FACE-TC group had a 3-fold odds of being in the high-congruence latent class (odds ratio [OR], 3.22; 95% CI, 1.09-9.57), compared with the dyads in the treatment as usual group. Statistically significant differences existed at 12 months (β [SE] = 1.17 [0.55]; *P* = .03]) (eTable 3 in Supplement 2) in the time 1 to time 4 model. White adolescents and families had significantly greater odds of membership in the high-congruence latent class than a small population of American Indian or Alaska Native, Asian, Black or African American, Hispanic or Latino, or multiracial adolescents and families (OR, 3.97 [95% CI, 1.07-14.69]; β [SE] = 1.38 [0.67]; *P* = .04) (Table 2; eTable 3 in Supplement 2). The association of 18-month outcomes with latent class membership by intervention was not significant and had an OR of 2.08 (95% CI, 0.92-4.69).

**Table 2.** Effect of FACE-TC on Latent Class Classification, Controlling for Covariates and Results of Logistic Regression Model for Time 1 to Time 4 (N = 117 Dyads)^a^

Variable	OR (95% CI)
FACE-TC	3.22 (1.09-9.57)^b^
Adolescent race and ethnicity	
White	3.97 (1.07-14.69)^b^
Other^c^	1 [Reference]
Adolescent age, y	
<14	1 [Reference]
14-17	1.74 (0.61-4.96)
Adolescent sex	
Female	1.85 (0.64-5.37)
Male	1 [Reference]
Family educational level	
<High school	1 [Reference]
High school diploma or GED equivalency	0.76 (0.23-2.53)
≥College or university degree	1.72 (0.38-7.71)
Family household income, FPL	
After 2016	1 [Reference]
In or before 2016	1.67 (0.42-6.66)

Abbreviations: FACE-TC, Family-Centered Advance Care Planning for Teens with Cancer; FPL, federal poverty level; GED, General Educational Development; OR, odds ratio.

^a^ Model was estimated using a 3-step method in Mplus, version 8.4, to deal with measurement errors in latent class membership estimation.

^b^ Statistically significant at α = .05.

^c^ Other race and ethnicity included American Indian or Alaska Native, Asian, Black or African American, Hispanic or Latino, and more than 1 race.

Adolescents in the FACE-TC group compared with those in the treatment as usual group were significantly more likely to have an advance directive in the EHR at study closeout (80% [60 of 75] vs 19% [8 of 42]; *P* < .001) (Table 3). Documentation of goals-of-care conversations in the EHR at study closeout was low and did not differ between groups (14% [10 of 72] vs 10% [4 of 41]; *P* = .76) (Table 3). Adolescents in the FACE-TC group vs the treatment as usual group had a 19-fold odds of having an advance directive in the EHR at study closeout (OR, 19.20; 95% CI, 6.84-53.88; *P* < .05).

**Table 3.** Effect of FACE-TC on Documentation in the Electronic Health Record of Any Advance Directive or Advance Care Planning Goals of Care Discussion at Study Closeout (N = 117 Adolescents)

Variable	No. (%)		χ^2^ *P* value
	Treatment as usual group	FACE-TC group	
**Any advance directive**			
No	34 (81)	15 (20)	<.001
Yes	8 (19)	60 (80)	
**ACP goals-of-care discussion before study**			
No	37 (90)	62 (86)	.76
Yes	4 (10)	10 (14)	

Abbreviations: ACP, advance care planning; FACE-TC, Family-Centered Advance Care Planning for Teens with Cancer.

Regression analysis found no significant differences in advance directives by adolescent age, sex, and race and ethnicity as well as family household income and educational level (eTable 4 in Supplement 2) or goals of care (eTable 5 in Supplement 2). eTable 6 in Supplement 2 provides descriptive statistics by sex and race and ethnicity.

### Deaths

Among the 7 adolescents who died, 6 (86%) had an advance directive in the EHR at study closeout. Five deaths occurred before completion of the protocol. Treatments that were received 30 days and 7 days before death and the last Statement of Treatment Preferences completed before death are detailed in eTable 7 in Supplement 2. Of the 3 adolescents in the FACE-TC group who died, 1 died before completing a Statement of Treatment Preferences and 2 endorsed the option to stop all treatments to keep me alive in every situation. There was good congruence between adolescents and their families. The 4 adolescents in the treatment as usual group who died endorsed the option to continue all treatments or were unsure with 1 exception: stop all efforts to keep me alive was selected if the adolescent was physically impaired. There was poor congruence in 3 of the 4 dyads.

## Discussion

To our knowledge, this trial was the first to focus on the sustainability of congruence about end-of-life treatment preferences between adolescents with cancer and their families, answering the critical question “Is pACP too early?”^47,48^ Compared with families in the treatment as usual group, families in the FACE-TC group had a greater than 3-fold odds of membership in the high-congruence latent class, accurately reporting their adolescents’ end-of-life treatment preferences over 12 months after the intervention. This finding replicates that of the FACE trial with adolescents^25^ and adults^26^ with HIV, who also had 3-fold the odds of being in the high-congruence group for 1 year after the intervention compared with the active control group. Pediatric ACP in the FACE model highlights the importance of family-centered ACP communication, which integrates communication with the clinician given that many young adults with cancer who complete the ACP document Voicing My Choices^4^ do not initiate an ACP conversation with their families (35%) or clinicians (91%).^49^

The decrease in the efficacy of FACE-TC at the 18-month assessment answers another timing question: follow-up is needed at 12 months. Results support pACP as an ongoing process of regoaling (defined as relinquishing one set of goals [eg, curing the condition] and pursuing a new set of goals [eg, maintaining the child's quality of life]),^50^ which prepares people for future just-in-time decisions.^51^ With respect to goal-concordant care, adolescents in the FACE-TC group were significantly more likely to give their families leeway to do what they believed was best at the time considering the adolescents' wishes compared with adolescents in the treatment as usual group.^52^ Clinical outcomes for the 7 adolescents who died during the trial were complex. Although the sample was small, dying adolescents in the FACE-TC group (n = 3) wanted to stop all treatments, which had good family congruence. Dying adolescents in the treatment as usual group (n = 4) were either unsure or wanted to continue all treatments, which had poor congruence in 3 of 4 dyads. Adolescents in the FACE-TC group chose to trust their family members’ judgment to help maintain the quality of their last days of life.

Interventionists entered into the EHR the advance directives of the adolescents. This structure and standardization ensured that not only were advance directives completed but also the institutional mechanisms for filing documents in the EHR were followed in a timely and correct manner. However, similar to the pilot trial,^29^ the documentation of goals of care in the present trial was lacking. Central, accessible, and standardized formats in the EHR and quality metrics are needed, such as care and communication bundles.^29^

We found a race and ethnicity–based difference in the sustainability of congruence about end-of-life treatment preferences that was independent of adolescents’ preferences for continuing life-sustaining interventions. Although no race and ethnicity–based differences in congruence were found at time 1,^52^ over time White adolescents were 4 times more likely to achieve congruence with their families than a small population of American Indian or Alaska Native, Asian, Black or African American, Hispanic or Latino, or multiracial adolescents. The importance of this finding cannot be overstated as it reflects a difference in the effect of the intervention. However, without qualitative data, we cannot explain how the intersection of racism, religion or spirituality, family dynamics, illness, and communication leads to this complex finding. Race and ethnicity–based disparities in pACP, at a minimum, depend on context, the racial and ethnic match of study personnel to participants, geography, and culture.^53,54,55,56^ Data collected in an inclusive fashion and further research with an intention to address the relationship between race and ethnicity and pACP are needed.

We believe this third trial of the FACE model continues to demonstrate the power of ACP conversations to maintain open communication between patients and their families. The findings contribute to the evidence base of best-practice recommendations to guide clinicians on the when, who, what, and how of end-of-life conversations with adolescents with cancer.^57^ There has been recent debate about the benefits of ACP.^58,59,60^ Pediatric ACP in the FACE model helps adolescents with cancer and their families make difficult decisions they may not otherwise discuss. Supporting adolescents in having a voice and enabling them to engage in difficult conversations are worth the time and effort.

### Strengths and Limitations

This study has some strengths. The findings confirm the results of 2 other trials of the FACE model,^25,26^ using a rigorous trial design,^61^ vigilant blinding of assessors, and high participant retention across a 2-year period, even though the studies were conducted in different patient groups and with different interventionists. This replication of findings provides confidence in the interpretation that the FACE-TC intervention can support long-term family knowledge of their adolescent’s end-of-life treatment preferences.

This study also has some limitations. The participation rate may introduce selection bias and affect generalizability. Randomization generally corrects for bias, and the participation rate was similar to that in adult dyadic longitudinal palliative care studies.^62,63^ One-fourth of the potentially eligible dyads declined participation because they did not want to talk about pACP. Honoring an adolescent’s right to defer end-of-life decisions to their family or physician is important in showing respect for their autonomy and should not impede program implementation. Generalizability was also limited by the small number of participants from racial and ethnic minority groups. Social desirability bias could have occurred with the face-to-face administration of study questionnaires. Persons who could not speak or understand English were excluded because the intervention required the signing of a legal document. The protocol has since been adapted for Spanish-speaking participants.^13^ The refusal of many male adolescents to participate is consistent with the adult literature, which has shown that male individuals are generally less likely to participate in clinical trials of ACP.^64^ The predominance of female family members reflects that, in general, women are more likely than men to be in caregiving roles.^65^

The results were generalizable to White female adolescents whose families reported having college-level education and access to tertiary children’s specialty hospitals. We do not know if adolescents with hematologic cancer were less likely to participate in ACP or complete an advance directive than adolescents with solid tumors, which has been found in adults.^66^ The results of this trial were based on a thorough analysis of the missing data to provide an estimate of end-of-life treatment congruence at 12 months after FACE-TC (eAppendix 1 in Supplement 2; Figure 1).

## Conclusions

This randomized clinical trial found that families’ knowledge of their adolescents’ end-of-life treatment preferences was sustained for 1 year after the FACE-TC intervention, suggesting the need for yearly follow-up sessions. Race and ethnicity–based differences in the sustainability of this congruent knowledge reflect the difference in the effect of FACE-TC and require further study. The findings contribute to the when, who, what, and how of pACP with adolescents with cancer.

## Notes

Supplement 1.
Trial Protocol

Supplement 2. **eAppendix 1.** Additional Details for Latent Class Analysis for Hypotheses 1 **eTable 1.** FAmily CEntered (FACE) Advance Care Planning Intervention **eAppendix 2.** Descriptive Statistics of the Congruence Measure by Latent Classes **eTable 2.** Selected Results of Longitudinal Latent Class Analysis (LLCA) for Congruence Between Patient and Family Over Time Regarding Treatment Preference in Four Medical Situations (N=117 Dyads) **eTable 3.** Effect of Intervention on Latent Class Classification, Results of Logistic Regression Model Using 3-step Procedure for the 4-time Point Data (T1-T4) (N=117 Dyads) **eTable 4.** Intervention Effect on Odds of Having Completed Any Advance Directive Documented in the Electronic Health Record at Study Close-out: Results of Logistic Regression (N=117 Dyads) **eTable 5.** Intervention Effect on Odds of Having Completed ACP Goals of Care Discussion Documented in Electronic Health Record at Study Close Out: Results of Logistic Regression (N=117 Dyads) **eTable 6.** Documentation in the Electronic Health Record of Any Advance Directive or Advance Care Planning Goals of Care Discussion at Study Close Out by Gender and Race **eTable 7.** Health Care Utilization 30 Days and 7 Days Prior to Death from the Electronic Patient Health Record Form (N=7 Adolescents)

Supplement 3.
Data Sharing Statement
