# Supplementary material for: Effect of the Family-Centered Advance Care Planning for Teens with Cancer Intervention on Sustainability of Congruence About End-of-Life Treatment Preferences: A Randomized Clinical Trial
Source: JAMA Netw Open. 2022 Jul 12;5(7):e2220696. doi: 10.1001/jamanetworkopen.2022.20696 (PMC9277499; doi:10.1001/jamanetworkopen.2022.20696)
Supplement: Supplement 1. — Trial Protocol [file jamanetwopen-e2220696-s001.pdf]

SPECIFIC AIMS. Building Evidence for Effective Palliative/End of Life Care for Teens with Cancer.

Pediatric Palliative Care and End-of-Life (pPCEOL) interventions are needed for the 400,000 children in the United States with Life Limiting Illnesses (LLIs),[1,2] particularly those with cancer as it is the leading cause of disease-related death for adolescents and young adults (AYAs). Survival has improved far less for 15 to 24 year-olds than it has for older patients,[3,4] and about one-fourth of children with cancer will die prematurely.[5,6,7] Early conversations about End-of-Life (EOL) treatment decisions hold the promise that quality care will increase for children living with LLIs, consistent with NINR's campaign, *Palliative Care: Conversations Matter*.[8] Avoiding these difficult conversations may contribute to serious adverse consequences such as inappropriate and unwanted costly care,[9] parents being charged with neglect,[10] court battles and even legislative intervention.[11] Families may be torn apart,[12] rather than strengthened by parents' desire to protect their children and to be a good parent.[13-20] Parents want to hear their child's own voice[21] and to understand their child's goals of care, values, and choices, but need help breaking the ice.[22] Such interactions could also address the *Healthy People 2020* goal of increasing the proportion of AYAs who are connected to a parent with whom they can talk about serious problems.[23]

Advance Care Planning (ACP), a key component of palliative care, is a process of preparation and skill development to facilitate discussions about future medical care choices.[24,25] With funding from the American Cancer Society, we developed and piloted tested FAmily CEntered ACP for Teens with Cancer (FACE-TC), a family intervention consisting of three weekly, 60 minute sessions involving: (1) completion of the Lyon ACP Survey (Adolescent & Surrogate versions), (2) participation in the Respecting Choices Interview, facilitating conversations about goals of care, and (3) execution of an advance directive. Conducted in a dyadic format with a certified facilitator, this developmentally/culturally appropriate intervention is informed by transactional stress and coping theory,[26] giving adolescent patients some control in a low control situation. (Surrogates are persons who would make decisions for AYAs, if they could not speak for themselves. For teens aged 14-17, only legal guardian(s) can be surrogates, whereas teens aged 18 up to 20 years choose a surrogate before ACP**.** Surrogates are referred to, from this point on, as family.[27] Our preliminary data demonstrated feasibility, acceptability, safety and initial efficacy of FACE-TC to increase congruence between adolescents and their families in treatment preferences,[28, 29] which should optimize patients' and families' quality of life.[30-32]

To test the efficacy of FACE-TC on key outcomes, we propose using an intent-to-treat, longitudinal, prospective, multi-site, randomized controlled trial (RCT) design. Adolescents with cancer, aged 14 up to 20 years, and their families (N=130 dyads; N=260 participants) will be recruited and randomized to FACE-TC or Treatment as Usual (TAU) control. They will complete standardized questionnaires at baseline and 3, 6, 12, and 18 months post-intervention. Our goal is to assess the extent to which FACE-TC helps AYAs with cancer and their families: (1) reach and maintain better congruence in treatment preferences over time; (2) improve their quality of life; and (3) document goals of care and advance directives earlier in the course of their LLI.

AIM 1. To evaluate the efficacy of FACE-TC on patient-family congruence in treatment preferences.

H1a: FACE-TC participants will better maintain congruence over time, compared to controls.

H1b: Development of congruence may not be homogeneous and FACE-TC may influence the pattern of congruence development.

AIM 2. To evaluate efficacy of FACE-TC on AYA patient quality of life [pain/fatigue, psychological, spiritual/existential (meaning/purpose, peace] and family quality of life (psychological, spiritual/existential).

H2: FACE-TC participants will report higher or greater improvement in quality of life, compared to controls**.**

Aim 3. To evaluate the efficacy of FACE-TC on early completion of pACP goals of care and advance directives, i.e. advanced planning of EOL care.

H3: FACE-TC patients will be more likely to have completed goals of care (e.g., Statement of Treatment Preferences) and advance directives (e.g., Five Wishes) than controls at 18 months post-intervention.

Exploratory Aim. Among the adolescents who die during the course of this study, we will explore if FACE-TC improved the match between patients' goals of care and the medical care received at the EOL.

The World Health Organization,[33] Institute of Medicine[34] and American Academy of Pediatrics[35] recommend that pPCEOL be an essential component of care from diagnosis to the EOL. Our ultimate aim is to develop an international model of structured pediatric ACP (pACP), as part of pPCEOL. Study results will exert a significant impact on service-delivery models of pPCEOL care in three ways: (1) by meeting the needs of children/families in preparing for the EOL, as set out in the IOM 2003 report, *When Children Die*;[34] (2) by building upon national guidelines for improving the psychological and spiritual/existential aspects of quality of care as a dimension of pediatric palliative care;[36] and (3) by providing an evidence base to meet practice guidelines[37,38] for an intervention that can be extended to other adolescent with LLIs.

RESEARCH STRATEGY

1. Significance

1.a. ACP enhances PCEOL care. FAmily CEntered ACP for Teens with Cancer (FACE-TC) is an empirically guided intervention aimed at improving pediatric Palliative Care and End-of-Life (pPCEOL) care by facilitating Advance Care Planning (ACP) among adolescent and young adult (AYA) patients and their families. ACP provides another level of support, rather than overlapping with or competing with the ongoing efforts of the palliative care team. Research has reported improved patient outcomes associated with EOL discussions[39] and with **Briggs' (Co-I)** & Hammes' Respecting Choices® Next Steps ACP intervention.[40] ACP interventions with adult patients demonstrate that when patient wishes are expressed in a living will and to a surrogate, congruence is high between patient preferences for care and the care actually received before death.[41-45]

Our pilot data suggest the potential for similar effects with FACE-TC. We conducted a small (N= 30 adolescent/family dyads), single site, intent-to-treat RCT with two study arms: FACE-TC or Treatment as Usual (TAU) control. Mean age of adolescents with cancer (n=30) was 16 years, 60% were male and 43% were African-American. Diagnoses were leukemia 47%, brain tumor 27%, solid tumor 20% and lymphoma 7%. During the study, seven adolescents died from cancer (4=controls; 3=intervention). In a post-hoc electronic medical record (EMR) review, we found that, on average, intervention patients completed an advance directive 325 days before death and first consulted with hospice 308 days before death, whereas the control patients, on average, consulted hospice 175 days before death and completed an advance directive 132 prior to death. Thus, palliative care services were utilized sooner for AYAs who participated in FACE-TC than for those who did not, indicating the FACE-TC intervention is consistent with the NINR mission to increase early palliative care through conversations about future care. This potential effect of FACE-TC is important because Temel,[46] among others,[47] demonstrated earlier palliative care improves survival and quality of life. Our study will fill the gap in knowledge of how AYAs with cancer may benefit from early pACP.

We also found significantly higher rates of congruence in treatment preferences among FACE-TC intervention AYA/family dyads, compared to treatment-as-usual (TAU) control dyads, as measured by the Statement of Treatment Preference (SOTP).[48] The SOTP is a decision aide to assist AYA's in verbalizing goals of care in situations involving potential complications that might occur as a result of the advanced cancer diagnosis; it allows the AYAs to verbalize acceptable and unacceptable outcomes and determine the extent of treatment preferences. As illustrated in Figure 1, families in the FACE-TC intervention (n=17 dyads) were more likely to know what their AYAs wanted for EOL care, compared to controls (n=13 dyads). In situations 1 and 2, control families thought their teens wanted the opposite of what their teen actually preferred. The proposed study will be the first to explore whether participation in a pACP intervention increases the likelihood that treatment received in the last week of life matches AYA patients' goals of care.

Figure 1

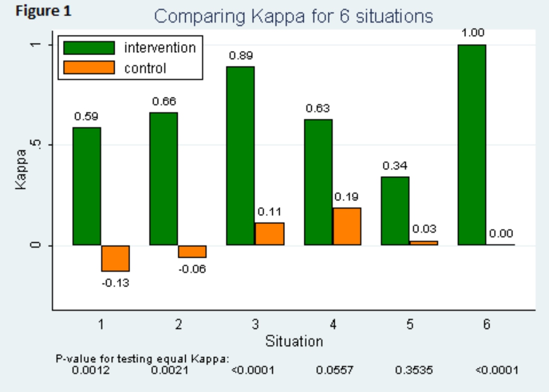

1.b. AYAs should be involved in ACP. Many adolescents are eager to participate in ACP, to talk about their goals and values regarding their own EOL care.[14, 49,50] However, despite professional recommendations, in practice, goals for shared decision-making with adolescents are often unrealized.[51-53] In a post-hoc survey of 24 members of the health care team treating patients in the FACE-TC pilot study, we found only 46.7% felt it was always appropriate to obtain an advance directive if the patient, aged 14-17, could comprehend and verbalize their wishes.[54] This increased to 80% if the adolescent was age 18 or older. This finding is congruent with a study of parents who were more comfortable communicating about pPCEOL the older their child with a life limiting illness (LLI).[55] Contrary to clinical and parent beliefs, empirical evidence supports the practice of allowing even young adolescents to make decisions about their own health. Research also indicates that children ages 14 years and older do not differ from adults in their competence to make informed treatment decisions.[56] Nor is there reason to suspect that young adolescents have a less mature understanding of death than those aged 18 or older.[14,34]

1.c. Need for evidence-based pACP interventions. In spite of adolescents' cognitive capacity and desire, only three pediatric ACP (pACP) programs are reported in the peer reviewed literature.[57] A qualitative study, Footprints, focused on patients aged 8-20 years with Duchennes' Muscular Dystrophy.[58] The second, developed by Hammes at **Gundersen**, one of our research partners, involved conversations between an ethicist and *parents* of children who were dying about the *parent's* goals for their child's care.[50] Only **Lyon's**

(PI) pilot study of FAmily CEntered ACP for Teens with HIV (FACE-HIV) employed a randomized controlled trial (RCT) to evaluate a structured pACP intervention. Forty (adolescents with HIV/family) dyads were randomized to FACE or a Healthy Living Control condition. Three weekly standardized sessions took place in an outpatient setting. Medical, psychosocial and spiritual goals of care were addressed. Main findings were increased congruence in treatment preferences, decreased decisional conflict and completion of advance directive documents with no adverse events.[22,59,60] In a recent systematic review of children's engagement in health related decisions, only the FACE-HIV pilot focused on supporting *children's participation* in decisions about their medical care *with their family*.[61]

Table 1. Percent Observed Agreement by Intervention Group and by Situation

Situation	Group	Specific Response Agreement: Continue treatment N (%)	Limit treatment N (%)	Don't know N (%)	Overall Response Agreement N (%)	Odds Ratio
1	Intervention	11 (64.7)	1 (5.9)	2 (11.8)	14 (82.4)	2.1
	Control	9 (69.2)	0 (0.0)	0 (0.0)	9 (69.2)	
2	Intervention	10 (58.8)	3 (17.7)	1 (5.9)	14 (82.4)*	10.5
	Control	3 (23.1)	0 (0.0)	1 (7.7)	4 (30.8)*	
3	Intervention	10 (58.8)	2 (11.8)	4 (23.5)	16 (94.1)*	13.7
	Control	7 (53.9)	0 (0.0)	0 (0.0)	7 (53.8)*	
4	Intervention	7 (41.2)	2 (11.8)	4 (23.5)	13 (76.5)	3.8
	Control	4 (30.8)	2 (15.4)	0 (0.0)	6 (46.2)	
5	Intervention	5 (29.4)	6 (35.3)	0 (0.0)	11 (64.7)	1.6
	Control	2 (15.4)	5 (38.5)	0 (0.0)	7 (53.8)	
6	Intervention	16 (94.1)	1 (5.9)	0 (0.0)	17 (100)	>20
	Control	10 (83.3)	0 (0.0)	0 (0.0)	10 (83.3)	

*Statistically significant at 0.05 level using Fisher's exact test

Situations

1. Long hospital stay, many treatments, chance of living thru complication low.
2. Cancer has spread and treatments will extend my life by no more than 2-3 months.
3. Expected never be able to walk or talk again, & need 24 hour nursing care.
4. Never know who I was or I was with and would need 24 hour nursing care.
5. Want cardiopulmonary resuscitation attempted unless my physicians deems otherwise.
6. "Mechanical ventilation"

1.d. pACP can improve pPCEOL care. Findings from our preliminary evaluations of FACE-HIV and FACE-TC suggest that this pACP intervention can improve pPCEOL care in several ways. First, with more patient-approved advance directives in place, there may be an overall reduction in aggressive EOL treatments that increase patient suffering as well as burdens on the healthcare system. We found that FACE-TC patients-families were significantly more likely to agree to limit treatments across six hypothetical "bad outcome" scenarios, as illustrated in Table 1,[29] which replicated results from our HIV pilot.[60] Second, this intervention can help reach the goal of Meaningful Use of EMR by increasing the documentation of advance care directives, ACP conversations and EOL care. The EMR can be a critical tool for communicating about ACP.[62-65] Goals of care and designated surrogate decision-makers should be present in the History and Physical as well as in the progress, consult, and discharge notes of all disciplines.[66] FACE-TC can facilitate Meaningful Use by encouraging input of advanced care plans into EMR, and EMR utilization can enhance PCEOL care by providing ready access to patients' goals of care, designated surrogate decision-makers and advance directives. In our pilot study, at the 3 month post-intervention wave, for intervention patients, the *Five Wishes*© advance directive and Statement of Treatment Preferences were easily found in the electronic medical record with secured copies sent by email to the treating physician [FACE-TC 15/15=100% vs. Controls 0/15=0%]. Third, this pACP intervention can address disparities in the use of palliative care by African-Americans[67,68] who historically have experienced discrimination by health care institutions[69] and may interpret discussion of limitations to care as an attempt to deny beneficial care.[67-69] This may account for the low levels of hospice care and use of Do Not Resuscitate orders the PI found among the 41 children who died during the Pediatric AIDS Clinical Trials,[70] and the underutilization of hospice and home health care services at the EOL for children with complex chronic conditions.[71] The proposed study can minimize disparities by providing equitable access to pACP for the approximately 30% of the patient sample who will be African-American. In the pilot FAC-TC study, all intervention families, 43% of whom were African-American, completed the *Five Wishes*© and received a copy with instructions about how to change it.[28] As families learned about the benefits of completing the *Five Wishes*©, many asked for copies for their personal use. Our results support recent adult research showing that a structured ACP process increases advance directives among adult racial and ethnic minorities.[42,72] Thus, this proposal has the potential to minimize health disparities and to improve public health by minimizing physical, psychological and spiritual suffering an important goal of public health.

1.e. Need for empirical evidence of pACP effects on key palliative care outcomes. Because our pilot data suggest positive effects of FACE-TC, and to build the evidence base for pACP interventions, we propose conducting a fully powered RCT testing the effectiveness in terms of key outcomes related to the National Consensus Project pillars of palliative care: physical (pain, fatigue), psychological (symptoms of anxiety, depression) and spiritual/existential (meaning/purpose, peace).[36] The structure and content of the intervention, which integrates essential elements of theoretically grounded programs including Next Steps ACP intervention,[40,41,48] is intended to increase patient-family congruence on treatment preferences and ensure completion of advance care plans, which should improve quality of life for both patients and families. This intervention process was largely developed by a research collaboration between **Briggs** and colleagues with Professor Karin Kirchhoff and the University of Wisconsin-Madison School of Nursing who introduced the

Respecting Choices faculty to the representational approach to patient education[73] and the Common-Sense Model of Illness Representation.[74-76] According to the Common Sense Model, an illness representation is the set of thoughts (whether medically accurate or not) that a person has about a health problem and has five dimensions: identity, cause, time line, consequences, and cure/control.[77,78]

1.f. Potential for extension of FACE-TC to other LLIs. Our proposed study builds on a foundation of empirical work indicating that this pACP intervention is effective for AYAs with HIV[22,59,60] and is feasible, acceptable, safe and potentially effective for AYAs with cancer.[28,29] FACE-TC is a developmentally and culturally suitable pACP intervention that could readily be expanded to patients of the same age range with other LLIs, nationally and internationally, furthering research in this field.

The Affordable Care Act offers opportunities for pACP programs to participate in the planning, development and implementation of new delivery models. If successful, FACE-TC will establish effective and efficient care for the most vulnerable patients and their families that aims to identify the steps necessary to match medical treatment to patient and family goals through improved access to quality pACP, consistent with the goals of the National Priorities Partnership.[79] We will have established on a larger scale that: (1) pACP can be done safely and feasibly with AYAs at more than one institution; (2) the pACP process can be "institutionalized" such that it is available across the board, thereby creating a standard of care intervention, rather than a "nice to have"; and (3) that quality of care can be measured and improved (by measuring goals of care and advance directives in the EMR), thereby accomplishing NINR's mission to build evidence-based quality PCEOL interventions. Moreover, providing a structured interaction that facilitates conversations between AYAs and families about EOL is innovative and can help achieve the *Healthy People 2020* goal of increasing the proportion of AYAs who are connected to a parent with whom they can talk about serious problems.[23]

2. Innovation

2.a. Clinical Innovations. The proposed FACE-TC intervention offers a novel methodology with 10 significant improvements to current clinical pPCEOL practice. FACE-TC (1) promotes an active, patient-centered, family-engaged informed decision-making process for pACP, (2) is the first structured and individualized pACP intervention for AYAs with cancer,[57] (3) integrates the evidence-based Respecting Choices® *Next Steps* ACP intervention developed by **Briggs** and colleagues at Gundersen Health System,[40,48] (4) utilizes a structured curriculum delivered by certified facilitators, (5) focuses on promoting QOL for both AYAs and their families, (6) improves upon advance directive documentation alone[80-82] by having a healthcare worker facilitate patient and family conversations about treatment preferences, then share them with the health care team, thereby ensuring pACP actions are taken,[83,84] (7) preserves the integrity of the parent-child relationship,[85] (8) is grounded in Leventhal's theory of self-regulation and patient illness representations,[73-76] as well as Folkman's and Lazarus's transactional stress and coping theory;[26,86] (9) respects individual differences, involving patients/families in treatment decisions at the level they prefer[87]; and (10) gives adolescents a voice. Our findings have the potential to become standard-of-care evidence-based practice, which can overcome critical barriers to progress in pPCEOL and ensure the highest quality of scientific knowledge.[28,29,88-90]

2.b. Research Innovations. Our current proposal furthers previous research by focusing on a new patient population, AYAs with cancer, and by utilizing a longitudinal, prospective RCT design that enables us to: (1) track if goals for care changed over time and if congruence was sustained, recently referred to as the "regoaling process;"[91] (2) determine if dyads who change goals have better outcomes (e.g., less anxiety, depressive symptoms) than those who maintain their original goals; (3) demonstrate sustainability and generalizability; (4) prevent bias by assuring Research Assessors and chart abstractors are blind to random assignment; (5) evaluate FACE-TC effects with respect to time-variant (e.g., religion/religious beliefs & practices) and time-invariant covariates (e.g., gender, race, ethnicity) on study outcomes; and (6) examine a core set of key outcomes that are measurable and valid across gender and ethno-cultural perspectives.

Moreover, using an innovative *person-centered* analytical method for longitudinal data, *growth mixture modeling (GMM)*,[92-96] our proposed research will overcome problems with traditional *variable-centered* methods, which may overlook special needs of sub-populations. GMM models do not assume homogeneity and enable identification of underlying subgroups comprised of distinct outcome trajectories. The purpose is to identify distinctive patterns of outcome development trajectories over time (e.g., congruence in treatment preferences between AYAs with cancer and families) and examine how the typology of outcome development differs between the FACE-TC and control groups, controlling for time-invariant covariates (e.g., gender, race ethnicity)[97,98] and time-varying covariates (e.g., religion). The application of this approach to pPCEOL is innovative and consistent with the recommendations of NINR, The Science of Compassion: Future Directions in End-of-Life and Palliative Care, to conduct longitudinal, trajectory-based studies of tested interventions.[99]

3. Approach

3.1. **Overview**. We propose a prospective, longitudinal, 2-arm RCT to test the efficacy of FACE-TC on key measurable outcomes through 18 months post-intervention. Dyads composed of AYAs with cancer and their families (N=130 dyads; 260 subjects) will be enrolled and randomized to either the FACE-TC intervention or Treatment as Usual (TAU) Control group at a ratio of 2:1 [N=87 FACE-TC dyads and N=43 TAU Control dyads], because pilot data demonstrated treatment benefit. Based on preliminary studies,[28,59,100] we estimate 30% attrition by the 18 month post-intervention assessment (20%-25% due to death/complications and 10% due to dropout).[28] Of the original sample of 130 randomized dyads (N=260 subjects), we estimate we will have full longitudinal data at 18 months post-intervention for 91 dyads (N=182 subjects). See Appendix A for protocol schema.

3.2. Study Population. Participants will be recruited from Akron Children's Hospital (n=203) and St. Jude Children's Research Hospital (n=317) pediatric oncology programs, which serve approximately 520 AYA patients (ages 14-20) per year in addition to approximately 40 newly relapsed patients per year with leukemia, lymphoma, solid tumors or brain tumors. AYAs with all malignancies, at any stage of illness, and with all prognoses will be eligible to enroll. AYA inclusion criteria are: (1) ≥14.0 years and <21.0 years at the time of enrollment; (2) not known to be developmentally delayed*;* (3) Beck Depression Inventory, Second Edition (BDI-II), Total Score < 26;[101] (4) primary language is English (as some of the questionnaires are only available in English and the signing of an advance directive requires proficiency); (5) intact mental status (MMSE score ≤ 18);[102] and (6) knows diagnosis. Family inclusion criteria are: (1) ≥ 18.0 years at the time of enrollment; (2) not known to be developmentally delayed (3) BDI-II Total Score < 26;[101] (4) primary language is English; and (5) intact mental status (MMSE score ≤ 18);[102] and (6) knows patient's diagnosis. Participants will also be screened for homicidality, suicidality and psychosis to ensure competency to engage in shared decision-making. Appropriate referrals will be given to potential participants as needed.

We estimate approaching 288 AYAs with cancer with approximately 50% declining or initially ineligible (e.g., cannot identify a surrogate). Of the remaining 144 dyads, an estimated 10% will be ineligible at screening. We will randomize 130 AYA/family dyads (260 participants). We estimate that 30% of participants will be African-American and 10% Hispanic or Latino, understudied populations. See **Human Subjects** for details.

3.2.a. Rationale for Site Selection. St. Jude and Akron Children's have established palliative care programs. Multiple sites are required to recruit sufficient numbers of AYAs with cancer to ensure adequate power and increase the generalizability of the findings, which is critical to translational science. Block randomization to condition by study site will control for idiosyncrasies of site-specific effects. Training on the protocol to certification, validation of implementation by video, and standardized procedures should minimize differences by site. Children's National will be the coordinating center for the study, using its expertise in training to the protocol, data management, analysis, etc. but not serving as a recruitment site. The pilot study resulted in the hospital administration at Children's National making a commitment to train palliative care staff to implement FACE-TC as standard of care, thus "contaminating" Children's National as a study site for a randomized trial.

3.2.b. Recruitment. Recruitment will use principles of inclusiveness. We recognize that stage of disease progression may impact enrollment and will take steps to accommodate AYAs with advanced disease (See **Human Subjects**)**.** We initially intended to wait a minimum of one month after diagnosis of cancer. However, in interviews with families and physicians, a consensus emerged to approach all AYAs/families regardless of time since diagnosis, but only after confirming with the treatment physician that there are no contraindications (e.g., isolation for a Bone Marrow Transplant, in pain). We will not recruit on day of diagnosis, nor anyone in the Intensive Care Unit. We will examine whether "time since diagnosis" has an impact on study outcomes. Dyadic/family studies pose a challenge, as sometimes the patient wants to participate but the family member does not, or vice versa.[103] We demonstrated a high level of recruitment of eligible dyads, 92% for the HIV pilot[54] and 72% for the cancer pilot,[26] comparing favorably with adult EOL interventions in which only 30-47% of eligible patients participated.[104-106] Each site's interdisciplinary teams are experienced, having recruited and retained AYA research subjects, using successful methods described by PCEOL researchers.[100,107-109]

Recruitment Procedures. After reviewing EMR, the site research assistant (RA) will send a list of potentially eligible patients to the treatment physician, who then decides who can or cannot be approached. The RA, not the treating physician, will contact the patient to see if there is interest in order to avoid any perception of coercion. At the time of recruitment, the AYA or family will complete a "Contact Information Sheet." Eligible patients will be contacted until recruitment goals are reached. The selection, roles and responsibilities, and preparation of families are detailed in the 3-day Facilitator Training section of **Human Subjects,** as are additional procedures for recruitment.

Consent and Enrollment. Participants will undergo written informed consent/assent using an IRB-approved

documents(**Appendix B Documents**) prior to participation in any study procedures or data collection. Study sites will maintain documentation of participants' consent according to institutional guidelines. Eligible participants will be enrolled and complete the baseline assessment followed by randomization.
Randomization and Blindness. Randomization will be at the level of the dyad. Allocation will be concealed from the RA-Assessor to prevent bias. Block randomization by study site will help control for site differences.
3.2.c. Retention. Study sites will utilize current effective procedures for longitudinal retention as well as incorporating any agreed upon across sites. For example, the central database will prompt RAs when follow-up visits are due. Using such procedures, our group experienced only 5% attrition in our 3-month HIV pilot[59] and 7% attrition in our 3-month cancer pilot.[28]

3.3. Intervention and Control Conditions

3.3.a. FAmily CEntered Advanced Care Planning. See Table 2 for session content. FACE-TC will provide an opportunity for the AYA and family to engage in a process of pACP that psychologically requires the dyad to acknowledge that life is finite and to think about QOL near the end of life.[149] AYAs and families told us that a one-session ACP intervention, as is often done with adults, was "too quick." They wanted more time to reflect and consult with significant others before signing a legal document. So using a Community Based Participatory Research approach,[28] we interviewed patients and their families, and conducted focus groups, to design the intervention. We then Beta tested the intervention with three cancer patient/family dyads to further refine the model. The curriculum based FACE-TC that adolescent patients and their families found most worthwhile, and which the Institutional Review Board approved, consists of: The Session 1 ACP Survey, originally developed for adults, which primed the dyad for the kinds of issues that would be discussed in Session 2; the Session 2 Respecting Choices Interview, which is theoretically grounded and empirically-tested; and the Session 3 Five Wishes advance directive, which is the document the legal department at Children's National had approved for use with our patients. Both pilot studies (FACE-TC & FACE-HIV) demonstrated that a three session intervention is acceptable and feasible with 100% attendance at all 3 sessions, scheduled weekly.[29,60]

Table 2: Description of FAmily CEntered (FACE) Advance Care Planning Intervention.

	Session 1	Session 2	Session 3
**Session Foundation**	Lyon Family Centered Advance Care Planning (ACP) Survey - Adolescent and Surrogate Versions © to set stage for EOL conversation.	*Next Steps: Advance Care Planning Respecting Choices Interview Interview®* (Briggs and Hammes, 2012-2013)	The Five Wishes © is a legal document that helps a person express how they want to be treated if they are seriously ill and unable to speak for him/herself. Unique among living will and health agent forms - it looks to all of a person's needs: medical, personal, emotional, spiritual.
**Session Goals**	**1. To assess the adolescents' values, spiritual and other beliefs, and life experiences with illness and EOL care.**<br>**2. To assess when to initiate ACP planning.**	**1. To facilitate conversations and shared decision-making between the adolescent and surrogate about palliative care, providing an opportunity to express fears, values, spiritual and other beliefs and goals with regard to death and dying**<br>**2. To prepare the guardian/surrogate to be able to fully represent the adolescent's wishes**	**1. Which person the teen wants to make health care decisions for him/her;**<br>**2. The kind of medical treatment the teen wants;**<br>**3. How comfortable the teen wants to be;**<br>**4. How the teen wants people to treat him/her.**<br>**5. What teen wants loved ones to know;**<br>**6. Any spiritual or religious concerns teen may have.**
**Session Process**	1. Orient family to study and issues.<br>2. Adolescent is surveyed privately;<br>3. Surrogate is surveyed privately with regard to what they believe their adolescent prefers.	Stage 1 assesses teen's understanding of condition;<br>Stage 2 explores teen's philosophy about EOL decision-making;<br>Stage 3 reviews rationale for future decisions teen would want surrogate to act on;<br>Stage 4 uses Statement of Treatment Preferences to describe scenarios/choices;<br>Stage 5 summarizes need for future conversations . Referrals are made.	For adolescents under the age of 18, the Five Wishes © must be signed by their legal guardian.<br>Processes, such as labeling feelings and concerns, as well as finding solutions to any identified problem, are facilitated. Appropriate referrals .<br>*These sessions may include other family members or loved ones.

Although no disagreements about EOL preferences emerged which could not be managed by the FACE-TC protocol, in our pilot study we developed a standard approach for managing unresolved disagreements. If the dyads' issues are unresolved, families will be referred to the site ethicist or site chaplain. (See details in **Human Subjects**).

3.3.b. Treatment as Usual Comparison Group, plus Advance Directive Information Booklet. To minimize the burden to ill AYAs, we have chosen a Treatment as Usual comparison condition. This group will be provided with an advance care planning booklet that encourages conversation with the primary treating physician. Existing practice is for an advance directive form to be handed to patients aged 18 years and older in the Emergency Department by a clerk or mailed to a teen on his or her 18th birthday. At Akron Children's Hospital patients are told that assistance with preparation of or changes to an advance directive may be obtained 24/7 through the chaplain-on-call. Although Akron Children's Hospital has no specific policy written to address the minor patient, the terms "patient (as appropriate and as desired)" are used throughout multiple hospital policies addressing decision-making and family-centered care. St. Jude

hospital policy is similar to the above with two additions. Guidance for completion of advance directive documents is done by social workers. For minors with LLIs pACP discussions are deferred to the physician.

3.4. Data collection procedures. Assessments occur at baseline, and 3, 6, 12, and 18 months post-intervention. At each site the assessments and intervention will be administered by a research team comprised of the site Co-I and two RAs (RA-Assessor & RA-Interventionist).

3.4.a. Visit protocol:

Screening Visit (~15 min): The RA-Assessor presents the AYA with cancer and family with an Information Sheet (**Appendix B**) describing the study and conduct an initial assessment about whether they are eligible for enrollment. After consent/assent (**Appendix B**), further screening for inclusion/exclusion criteria is conducted.

Baseline Visit (~60 min): At enrollment and prior to randomization, baseline measures will be obtained. Entry of baseline data by the RA-Assessor will trigger computerized randomization of patient/family dyads to either FACE-TC intervention or TAU Control using a randomly permuted block design and a 2:1 ratio by study site. The data base system will then notify the RA-Interventionist who will schedule the next study visit.

The AYA and family will learn their assignment when the RA-Interventionist calls to schedule study sessions. Following randomization, intervention dyads will be scheduled for 3 study sessions, scheduled approximately one week apart. Attendance will be recorded to assess effects of full vs. partial participation in FACE-TC.

Follow-up Visits (~60 min each): RA-Assessor will obtain follow-up measures from the AYA and family at 3, 6, 12 and 18 month post intervention. See **Appendix A** Protocol Schema for timing of measure administration.

3.4.b. Measures. The selected measures have demonstrated adequate to good reliability, validity and appropriate norms (See **Appendix C**). They will be administered separately to the AYA and family in a private room by the RA-Assessor who is not the interventionist and is blinded to treatment assignment.

Primary Outcomes**:** (See Table 3 for timing of measure administration.)

Statement of Treatment Preferences (SOTP)[40,48,110] is a decision aid and communication tool. The SOTP will be used to document goals of care in “bad outcome” situations and the families’ understanding of what the AYA would want. It is to be entered into the patient’s EMR as an addendum to the Five Wishes. SOTP provides the data for the primary outcome of the study, congruence in treatment preferences over time. SOTP was used with AYAs in FACE preliminary studies.[28,29,59,60]

Five Wishes© (Towey, Aging with Dignity Component developed with support from The Robert Wood Johnson Foundation) is a legal document that facilitates having people express their treatment preferences if they were seriously ill or unable to communicate their wishes. The adolescent will complete this along with their family during the third intervention session. *Five Wishes©* is legally sufficient for those over the age of 18. It can also serve as a tool to facilitate the participation of younger AYAs in shared decision-making. Wiener and colleagues[111,112] found the *Five Wishes©* helped teens with cancer and was developmentally appropriate, consistent with our pilot data. A newer tool, *Voicing My Choices*,[111] is under development. For AYAs under age 18, the *Five Wishes©* must be signed by their parent or legal guardian to be legally sufficient. One study outcome will be the completion of *Five Wishes©* or other advance directive standard for the setting/study site in the electronic medical record at each data collection wave. See **Appendix D**.

Patient-Reported Outcomes Measurement Information System (PROMIS[113-115]) is a self-report developed for use with pediatric cancer patients to assess health-related quality of life. AYAs will complete the short forms to provide brevity and flexibility/portability. Emotional Distress-Anxiety; Emotional Distress-Depressive Symptoms; Fatigue; and Pain Interference forms each have 8 items. This measure is theoretically grounded and demonstrates feasibility, acceptability and validity in measuring pediatric patient-reported outcomes. Although normative data are available only for AYAs up to age 18 years, for comparison purposes we will use PROMIS with 18-20 year old patients with cancer. This will provide AYA measures of anxiety/depressive symptoms.

Beck Depression Inventory-II (BDI-II)[101] is a 21-item scale self-report measure to assess presence of symptoms of depression and severity of symptoms reported. This instrument has shown a high content, construct, and factorial validity. Reliability and validity are good. This will provide family measure of symptoms.

Beck Anxiety Index (BAI)[116] is a 21-item measure assessing severity of subjective, somatic, and panic-related symptoms of anxiety. Respondents rate the degree of discomfort experienced as a result of each symptom over the past week on a 4-point Likert scale (range 0-3). Test-retest reliability for this measure is acceptable ($r = .75$) as is internal consistency reliability ($\alpha = .92$). This will provide family measure of symptoms.

Spiritual Well Being Scale of the Functional Assessment of Chronic Illness Therapy – Version 4 (FACIT-Sp)[117] is a 23-item scale focusing on the existential aspects of spirituality as one aspect of quality of life, i.e., spiritual growth. We have chosen this scale in part because it makes no reference to God and will therefore be more culturally sensitive to those with non-theistic beliefs. There are two subscales: meaning/peace and faith. Items

cover issues such as: having a reason to live, finding meaning in one's life and finding peace. Validation samples were outpatients with HIV and cancer. It is used to study spirituality in teens with chronic illnesses.[118]

Time-Varying Covariates:

The *Brief Multidimensional Measurement of Religiousness /Spirituality (BMMRS-adapted)* [119-123] for use in health research will be used to assess the construct of religion and religious beliefs for both AYAs with cancer and their family and its impact on EOL treatment preferences. [124-125] Variables of interest include: religion (none, don't know/religious preferences) and Daily Religious Experiences (e.g., feeling God's presence).

Time-Invariant Covariates are age, gender, race, ethnicity, time since diagnosis, education, socioeconomic position, and study site.

Exploratory Outcome:

Medical Record Chart Abstraction in Cerner and Epic will be conducted to document the following variables in the final week of life: date and location of death, referral to hospice, hospital admissions, emergency department visits, mechanical ventilation, resuscitation, advance care directive, surrogate decision maker identified, documentation of conversations about goals of care (e.g., Statement of Treatment Preferences, resuscitation preferences, Do not Resuscitate or Allow a Natural Death orders, documentation of the Next Steps discussion using standardized template), and chemotherapy. We will be relying on our experts, **Dr. Watson and Ms. Briggs**, to develop a standardized data extraction sheet for the study variables of interest, and to identify key elements of pPCEOL.[126] Chart extraction will be conducted by a researcher blinded to randomization to study arm to prevent bias. A second researcher also blind to randomization will provide a reliability check. Differences will be reconciled by consensus between the two researchers.

Measures	Participant	Time Point	Description
Demographic Data Interview	Patient Family	Screen	Demographic data collection, including diagnosis and staging
Medical Chart Abstraction	Chart Abstraction	BL, 3, 6, 12, 18	Diagnosis, time since diagnosis, hospitalization, ER visit or other treatments since last study visit, care coordination
Psychological Interview	Patient Family	Screen	Standardized questions to screen for sucidality, homicidality and psychosis
Beck Depression Inventory-II	Patient Family	Screen, 3, 6,12, 18	Used as a screening tool for both AYA and family. Used to assess depressed mood during post-intervention assessments for family only. 21 items.
Statement of Treatment Preferences (STP)	Patient Family	BL, 3, 6, 12, 18	Tool to express values and goals related to future decision-making re: frequently occurring situations. 6 situations.
PROMIS Short Forms	Patient	BL, 3, 6, 12, 18	Valid and reliable measure of patient's Physical Function Mobility; Physical Function Upper Extremity; Emotional distress-Anxiety; Emotional Distress-Depressive Symptoms; Fatigue; and Pain Interference. 8 items each.
Brief Multidimensional Measurement of Religiousness/Spirituality (BMMRS-adapted)	Patient Family	BL, 3, 6, 12, 18	Assess the construct of spiritual functioning and religious practices, e.g. religious preferences and practices, feeling God's presence. Nonreligious participants can pass on these items. 38 items.
Brief-Religious Coping Questionnaire (Brief RCOPE)	Patient Family	BL, 3, 6, 12, 18	Assesses positive and negative religious coping methods. Study will use 14-item version
Experience of death of loved one to cancer	Patient	BL, 3, 6, 12, 18	Single item
Quality of EOL Communication Questionnaire	Patient Family	End of Session 1,2,3	Process measure assessing quality of communication between Patient/Family and the interviewer. 5 items.

3.5. Study Coordination and Operational Plan

3.5.a. Investigators and Research Team. The PI, Dr. **Lyon**, is a clinical health psychologist who brings her unique expertise in adapting ACP to adolescents with cancer and HIV/AIDS and to adults with AIDS. Her successful research on pACP includes two ongoing RO1 multi-site PCEOL investigations. In the recent pilot study of FACE-TC all predetermined benchmarks were exceeded: enrollment of 72% of eligible families, 100% attendance at all three sessions, 93% retention, and 100% of families rating FACE-TC as worthwhile,[28,29] replicating the pilot FACE-HIV study.[22,59,60]Ms. **Briggs**, MA, MS, RN (Co-I, Gundersen) has been a Co-I with Dr. Lyon on the ACP studies. She is a core member of our team and brings her unique expertise in the development of Next Steps ACP program. She will contribute her conceptual and research experience.

Dr. Sarah **Friebert** (site Co-I, Akron Children's) is a leader in pPCEOL, including informed decision-making for AYA patients, integrating pediatric palliative care into pediatric hematology/oncology and hospice care. She is the Director of Pediatric Palliative Care at Akron Children's Hospital. In 2012 her Pediatric Palliative Care Center received the Circle of Life Award from the American Hospital Association recognizing its innovations in pPCEOL. Dr. Justin **Baker (**Co-I, St. Jude) is the Chief of the Division of Quality of Life and Palliative Care at St. Jude Children's Research Hospital. Dr. Baker has been involved in making recommendations for palliative care for children with terminal cancer and implementing and evaluating policies related to palliative care referrals. Dr. Baker has developed a model of Individualized Care Planning and Coordination which includes family-centered ACP and ethical decision making for AYAs with cancer. Drs. Friebert and Baker**,** recognized leaders in research with pPCEOL, will provide oversight for daily operations in their respective hospitals. They will monitor accrual, scheduling, consent, patient safety, clinical assessments, referrals, and data validity. They will meet with the research staff weekly for this purpose. They will nurture an Oncology Fellow who will be introduced into the field of PCEOL research.

Dr. **Harold Koenig** is a physician and nurse who is collaborating with Dr. Lyon currently. Dr. Koenig's role will be a consultant assisting with the analysis, interpretation and dissemination of study findings with respect to spirituality and religion. Dr. Pamela **Hinds** (Children's Co-I)**,** an internationally recognized expert who is currently consulting with Dr. Lyon, will contribute nursing expertise in pediatric EOL studies and the needs of children and parents at the EOL, especially sensitivity to the child's voice. Dr. Anne **Watson** (Children's Co-I) is an expert in quality metrics extracted from EMR. She will have primary responsibility for gathering study metrics for determining if goals of care matched care received in the final week of life. Dr. Watson is a pediatric registered nurse with a Ph.D. in developmental psychology and an expert in use of EMR software across health care systems. Dr. Watson will help us to create a standardized chart extraction measure for EMR data to ensure reliability, validity and unbiased data collection with respect to examining patients' goals of care with care received in the last week of life.[126] She will also assist us with the analysis and interpretation of our exploratory outcome. Dr. **Wang** (Children's Co-I) contributes biostatistical expertise in GMM studies and experience with the PI's FACE studies. Dr. Anne **Kazak** will join us as the Chair of our Safety Monitoring Committee. Dr. Kazak is a clinical psychologist and expert in evidence-based interventions to promote competence in families facing the adversities associated with childhood cancer.

3.5.b. Administrative Structure. (See **Resources**) Dr. **Lyon** (PI) will have overall responsibility study oversight, including scientific and clinical leadership. In defining the structure and functioning for this study there are two conceptual components: the interventions (FACE-TC and TAU) and the assessments/data collection. A Research Coordinator will be responsible for coordinating all of the logistics and details between these two components across sites. Dr. **Lyon** will have primary responsibility for practical issues in training and implementing the intervention. Ms. **Briggs** and Dr. **Lyon** will be involved in the intervention training and ongoing assessment of performance. Each site Co-I will be responsible for all site activities and managing two research assistants (RAs) and one Fellow.

3.5.c. Site Roles and Responsibilities. Site Co-Is will oversee site activities and provide weekly, face-to-face supervision of the RAs. They are responsible for recruitment, retention, safety, fidelity to protocol, and supervision and support of RAs. The RAs will assist with recruitment, screening, enrollment and baseline screening measure collection, as well as the day-to-day functioning. RAs will be blind to random assignment. RA-Interventionists will be trained to implement the protocol. Only the RA-Assessor will be permitted to administer post-randomization assessments. The central site, Children's National, will serve as the data coordinating center and will be responsible for database design and maintenance and the statistical analyses. Sites will be overseen by a Safety Monitoring Committee (SMC).

3.6. Cross-Site Study Preparation

3.6.a. Investigator Meeting. A 2-day Investigator Meeting will be held in Washington, DC and will include all site Co-Is, RA-Assessors and consultants. The protocol, its scientific rationale, underlying ethics issues, implementation including recruitment and retention, will be reviewed with the entire team. Dr. Kazak will give a brief presentation in her areas of expertise. We will finalize the protocol, soliciting input from all sites, and update the FACE-TC Manual of Operating Procedures accordingly. The experienced Research Coordinator will organize and coordinate the Investigator Meeting.

3.6.b. Institutional Review Board (IRB) approval. The study sites, and the Coordinating Center, Children's National, will submit this protocol for IRB review prior to the funding decision to facilitate protocol implementation, if funding is awarded. If funded, the protocol will be circulated to and approved by the Safety Monitoring Committee. Protocol amendments will then be submitted to the site IRBs. If IRB approval fails, we will respond to concerns by amending the protocol accordingly.

3.6.c. Training Meeting. In month 9 of Year 1 there will be a 3-day training meeting of the RA-Interventionists and RA-Assessors on the intervention, which site-Co-Is will also attend. RA-Assessors will attend only one day of this training in order to maintain blindness. In the event of staff turn-over, a training session will be organized to provide in-person training by Dr. Lyon on all measures, study procedures, and the FACE-TC (See **Appendix D for Sessions 1, 2, 3 for protocol**) or TAU protocol.

Days 1 and 2 RA-Facilitator Training: In the first day of training in Washington, DC, the Next Steps ACP Facilitation Certification Course (Session 2, Next Steps: Advance Care Planning-Respecting Choices Interview**)** will be taught by certified faculty, Ms. **Briggs** and Dr. **Lyon**. This comprehensive skills training program includes pre-course materials (e.g., online ACP course, Next Steps-ACP manual), a classroom certification course, and validation of competency through review of individual video role-play demonstrations by Ms. **Briggs** and Dr. **Lyon**. RA-Interventionists are required to attain certification to criteria in delivering the Next Steps-ACP interview. Dr. **Lyon** will provide supplemental training at a later date if needed. RA-Interventionists must be certified to administer the intervention. A Booster Session, after 6 months or after the completion of 5 dyads randomized to intervention at each study site, will be conducted with Dr. Lyon and Ms. Briggs to reinforce success and share effective strategies for maintaining fidelity to the protocol.

Day 3 Training will orient all RAs to the study, the protocol, and standardized procedures for recruitment, screening and enrollment, including role plays. RA-Assessors will be trained by the Research Coordinator to conduct the screening, baseline and follow-up assessments to prevent process interviewer bias in the data collection. Training will also be provided on study logistics, data collection, regulatory issues and communication with the Data Center at Children's National.

3.6.d. Site Initiation. To begin screening/enrollment (1) the protocol must be approved at each site's IRB and all personnel must be certified in Human Subjects Research training, (2) personnel are recruited and trained for RA roles, and (3) each RA-Interventionist must complete certification as a Next Steps-ACP Facilitator. Dr. Lyon and the Research Coordinator will verify that the site has all components in place for the logistics of screening, enrolling, scheduling, performing assessments, administering interventions, and collecting the data.

3.6.e. Communications. Dr. **Lyon** is responsible for facilitating all communications regarding the project and for interactions with clinical trial sites. Communications will be primarily by teleconferences, telephone calls, and email. Dr. **Lyon** will have at least monthly contact (more frequent during study start-up) with staff at each site and quarterly teleconferenced booster sessions with RAs, specific to their roles. Goals are to facilitate communication and to monitor recruitment and retention of participants, adherence to protocol, identification of issues regarding data collection, and to address any concerns or questions. Identified success and/or potential barriers will be addressed, and action will be taken to resolve any barriers that arise during the study. Meeting minutes and Benchmarks will be kept by the Research Coordinator and circulated to all project staff.

3.7. Monitoring of Trial Conduct. To assure continuous quality for the intervention and its evaluation, monitoring will be ongoing.

3.7.a. Intervention Fidelity. Dr. **Lyon** and Ms. **Briggs** will review the first 5 DVD/audio recordings of intervention sessions from each site to ensure fidelity with the protocol. Thereafter, they will randomly review 1 DVD per week, rotating sites. Dr. **Lyon** and Ms. **Briggs** will use a competency checklist.

3.7.b. Procedure Monitoring. Dr. **Lyon** and Research Coordinator will monitor ongoing site IRB approval documentation and assist sites in annual continuing reviews. The Research Coordinator will keep copies of all regulatory forms, including consent-stamped templates from each site and staff members' Human Subjects Research Training approval certificates. Dr. **Lyon** and the Research Coordinator will perform twice yearly site monitoring visits while the intervention is being implemented to assure standardization of procedures and resolve any problems that are identified. They will review and confirm that all consents have occurred properly at the sites (via signatures, date and time) and that the sites are maintaining all participant and regulatory data.

3.7.c. Data quality monitoring. The REDCap (http://project-redcap.org) database from the FACE-TC pilot will be updated and expanded for this study, and the systems for data entry will be revised to address multi-site implementation. See **Resources** for details about the Children's National database system.
3.7.d. Safety Monitoring Committee (SMC). An external SMC will be assembled by Dr. **Lyon** in compliance with the Department of Health and Human Services. The SMC will have the responsibility of reviewing safety information, study progress, and other relevant data. The SMC will be chaired by Dr. **Kazak**, PhD. The SMC will meet a minimum of once a year. Duties will include review of any serious adverse event (SAE) reports, recommending follow-up or further action to the PI. The SMC will be notified of SAEs on an ongoing basis.
3.7.e. Benchmarks for success anticipated to achieve aims. Benchmarks for success will be (1) recruitment of half of the eligible population, (2) enrollment of 130 dyads for randomization, (3) retention of 80% of dyads by 3 month post-intervention, (4) 70% by 18 month assessment (N=91 dyads; 182 participants); (5) 90% data completion, and (6) <30% attrition. Any lags will trigger identification of barriers and solutions.
3.8. Analytical Plan. A variety of statistical methods, including descriptive statistics, logistic regression, latent growth model (LGM), and growth mixture model (GMM) will be used for data analysis in order to achieve the proposed analytical aims. Prior to parametric testing, scale reliabilities for multi-item measures (e.g., pain/fatigue, child and parent psychological, spiritual/religious measures) will be assessed using Cronbach's α and their composite scores will be used for data analyses.
3.8.a. Analytical Plan for AIM 1. *To evaluate the efficacy of FACE-TC on adolescent-family congruence in treatment preferences.* Congruence in decision-making for medical treatment will be tested based on agreement (i.e., both patient and his/her family choose the same option) on the *Statement of Treatment Preferences* in six different cancer-related situations. Kappa coefficients will be applied to assess chance-adjusted agreement between patient and family responses. Change in Kappa coefficient (congruence improvement) from baseline to each follow-up time point during the study period will be tested using bootstrapping technique.[127,128] The latent growth model (LGM) with categorical outcome will be used to test Hypotheses **H1a**, i.e., FACE-TC participants will have a higher congruence rate over time.[129-131] In the LGM, we will set time scores, except those for identification purpose, as free parameters to let the shape of growth trajectory be determined by data.[132] As such, the congruence development trajectory would have an empirically based nonlinear shape, instead of assuming a linear or nonlinear polynomial function (e.g., quadratic or cubic). We will apply the growth mixture model (GMM)[133-135] to test heterogeneity of congruence development trajectories and identify possible patterns of congruence in development trajectories in the full sample. Bayesian Information Criterion (BIC), Akaike Information Criterion (AIC), Lo–Mendel–Rubin likelihood ratio (LMR LR), the adjusted LMR LR (ALMR LR) test, and the bootstrap likelihood ratio test (BLRT) will be used for model comparison and identifying the optimal number of trajectory groups in the GMM. The latent class variable estimated from the GMM captures the pattern of congruence development trajectories. Time-invariant covariates (e.g., demographics including gender, race, ethnicity) will be used to predict the memberships of the latent trajectory groups; and time-varying covariates (e.g. religion/religious beliefs using Brief MMRS) will be included to predict the level of congruence at different time points. To test Hypotheses **H1b**, we will regress the latent growth slope factor and the latent class variable on FACE-TC intervention, controlling for covariates in the GMM to assess 1) how FACE-TC would affect the membership of the latent classes of congruence development; and 2) how the effect of FACE-TC on congruence change over time varies across the latent trajectory classes.
3.8.b. Analytical Plan for AIM 2. *To evaluate efficacy of FACE-TC on AYA quality of life[pain/fatigue, psychological, spiritual/existential (meaning/purpose, peace)] and family QOL (psychological, spiritual/existential).* The LGM and GMM models proposed for evaluating Aim 1 can be readily applied to evaluate Aim 2 and test Hypotheses **H2** where the outcome measures are continuous variables. When examining the effects of FACE-TC on QOL for AYAs with cancer (PROMIS measure of pain/fatigue, anxiety depressive symptoms, FACIT-SP) and their families (Beck depression/anxiety measures, FACIT-SP), socio-demographics will be controlled as time-invariant covariates, while time-varying covariates (e.g., religion/religious beliefs using Brief MMRS) will be included in the model to predict measures of QOL at different time points. In addition, family anxiety/depression measured at the end of the study period (i.e., 18 months post-intervention) will be included as a distal outcome in the GMM models, and how this distal outcome is associated with the patterns of the developmental trajectories of AYA QOL will be assessed. As the same model will be used to evaluate multiple outcomes, Bonferroni correction will be applied to exert a stringent control over false discovery.[136]

The LGM and GMM models will be implemented in Mplus.[137] When running GMM, we will check the class allocation consistency in the models to ensure that class membership remains basically unchanged after

covariates and/or distal outcome are included. Otherwise, the 3-step procedure will be employed to take into account the measurement error in most likely class membership.[138] If the model produces good class classification (e.g., Entropy[illegible]0.80), then the estimated latent class membership can be saved as a new variable and merged with the original data set; as such, further statistical analyses can be more flexibly conducted using the regular statistical packages like SAS, Stata or SPSS.[139]

As attrition is inevitable in longitudinal studies, robust model estimator (e.g., MLR) using the full information maximum likelihood (FIML) will be used for model estimation. Importantly, missing at random (MAR), instead of missing completely at random (MCAR), can be assumed in MLR. MAR is a plausible assumption that allows missingness to be dependent on observed measures like intervention assignment (e.g., participants in the control group may be more likely to drop out).

3.8.c. Analytical Plan for AIM 3. *To evaluate the efficacy of FACE-TC on early completion of pACP goals of care and advance directives*. First, we will use the two-proportion z-test to test the differences in proportions of completion of pACP goals of care and advance directives between FACE-TC and control groups. Then logistic regressions will be used to test the Hypothesis **H3**, controlling for socio-demographics. Interaction between intervention and ethnicity will be included in the models to test ethnic disparity in regard to intervention efficacy.

3.8.d. Analytical Plan for Exploratory Aim. We will explore if FACE-TC improves the match between patients' goals of care and the medical care received at the EOL among the adolescents who may die (about 25% of the sample). Descriptive statistics will be used to estimate the frequencies of the study variables. Chi-square statistics with Fisher Exact tests will be used to assess the difference in the match between FACE-TC and controls; and exact logistic regression will be applied to examine the effect of FACE-TC on such a match, controlling for covariates.

3.9. Sample Size and Power Analysis. Usually, the power of a statistical test depends upon significance level, sample size, and effect size of the exposure variable.[140] However, in longitudinal studies, the number of repeated measurements plays an important role in statistical power because there is a tradeoff between the sample size and the number of repeated measurements.[141] The statistical power of longitudinal data analyses is, therefore, strengthened by the presence of up to five repeated measures in our proposed study. For continuous outcomes with a modest observation autocorrelation (ρ=0.20) and moderate effect size (Δ=0.35), the estimated sample size to achieve a power of 0.80 at α=0.05 level and detect a moderate effect size (Δ=0.35) is about N=76 individuals at each of the 5 observation time points. For binary outcomes, a sample size of N=70 can achieve a power of 0.80 to detect a moderate response probability difference of d=0.17 given ρ=0.20.

According to our pilot study, the difference in rate of congruence between the intervention and control groups was about (83.4%-56.2%)=27.2% on average in all situations among AYAs with cancer. As such, assuming up to 30% attrition, our proposed sample of N=130 dyads will ensure a large enough statistical power for our proposed longitudinal analyses on patient data and parent data, respectively. For the cross-sectional logistic regression model proposed to evaluate Aim 3, a sample size of N=100 would achieve a power of 0.83 at α=0.05 level to detect an odds ratio of 4 in regard to having a Statement of Treatment Preferences or completion of advance directives (e.g., Five Wishes). The results of our pilot study show that everyone in the intervention group had completed Five Wishes, while almost none in the control group had done so, indicating that the corresponding odds ratio is much larger than 4. Therefore, our proposed sample size of 130 patients and 130 parents will ensure a large enough power for evaluating Aim 3.

3.10. Timeline. See Table 3.

**Table 3. Brief Timeline**	Year One				Year Two				Year Three				Year Four				Year Five			
	M 1-3	M 4-6	M 7-9	M10-12	M 1-3	M 4-6	M 7-9	M 10-12	M 1-3	M 4-6	M 7-9	M 10-12	M 1-3	M 4-6	M 7-9	M 10-12	M 1-3	M 4-6	M 7-9	M10-12
Finalize Protocol, IRB Approvals	x	x																		
Finalize Manual of Operating Pro.		x																		
Investigator Meeting		x																		
Protocol Review by SMC		x			x				x				x				x			
Finalize Database			x																	
Train Project Staff			x																	
Recruitment/Screening/Enrollment					x	x	x	x	x	x	x	x	x							
Project Oversight/Conference Calls			x	x	x	x	x	x	x	x	x	x	x	x	x	x	x	x	x	x
Monitor Sessions for Fidelity					x	x	x	x	x	x	x	x	x							
Site Monitoring/Data cleaning					x	x	x	x	x	x	x	x	x	x	x	x	x	x	x	x
Conduct Data Analysis						x		x		x		x		x		x	x	x	x	x
General Final Report																				x
Dissemination in Journals				x				x				x				x	x	x	x	x

3.11. Challenges and Future Directions. The greatest challenge in end-of-life, as well as dyadic studies, is recruitment and enrollment. If enrollment is below projections we will add another site to our study. As in the past, we will also provide booster training with role plays for effective recruitment. Interventionists will only be hired if they have flexible schedules to meet dyad's needs and are able to meet with families at home or in the hospital. We will also be using Fellows as back-up certified interventionists to provide another level of flexibility to meet the scheduling needs of families. Site providers' perspectives will be respected and addressed.[142,143] FACE-TC, if effective, will provide diligent, consistent, structured and standardized pACP in three ways: 1) by following through from ACP conversations to completion of advance directive documents; 2) by communicating a summary of these conversations and ACP documents to the health care team via HIPPA compliant email; and 3) by placing these conversations about goals of care and documents in the electronic medical record for easy access. If the proposed trial is successful, we plan to partner with Respecting Choices® Next Steps ACP program to translate this science into clinical practice conducting implementation and effectiveness research through the following process: 1) consultation in building the necessary systems to support pACP documentation and storage and retrieval of advance directives; 2) consultation in determining the resources necessary to embed this pACP service into healthcare delivery for appropriate patients/families; and 3) a competency-based facilitator certification program as a platform to train qualified professionals in the skills of having pACP conversations. We are honored to learn our FACE-TC pilot received "Initial Approval" for inclusion in the Research-Tested Intervention Programs (RTIPs) website developed by NCI and SAMSHA to translate evidence based programs into practice. Even though our intervention is not ready for "prime time" due to its small size, it is under consideration to address the growing need for evidence-based tools in this area. **Resource Sharing Plan.** The FACE-TC protocol will be available to the public over the Children's National web and, if approved, through RTIPS for immediate translation of this science into clinical practice, if our results prove successful and the protocol meet criteria. Study data will also be available upon request from Children's National's Department of Biostatistics and Informatics.

PROTECTION OF HUMAN SUBJECTS FROM RESEARCH RISK

1.Risks to Human Subjects

Human Subjects Involvement and Characteristics

We will be studying adolescents and young adults (AYAs) with cancer between the ages of 14 up to age 20, and their legal guardians (if under the age of 18) or their chosen surrogated decision-maker (if 18 years of age or older). We are not including patients from the coordinating center, Children's National, because the Children's National oncology staff who participated our American Cancer Society pilot asked to be trained/certified to the FACE-TC protocol and that training was provided. Thus, to prevent contamination of the randomized design, Children's National will not be eligible to participate. We anticipate having 520 potentially eligible patients from St. Jude and Akron Children's, as demonstrated in Table 4. We will recruit for approximately two years three months until we enroll 130 dyads who will be randomized at a ratio of 2:1 into either the FACE-TC intervention group (n=87 dyads) or TAU Control group (n = 43 dyads) with the goal of

obtaining data from approximately 91 dyads at 18 month post-intervention for statistical analysis. We estimate that approximately 40% of the sample will be minorities (African-American, Hispanic/Latino or Asian). A discussion of selection of guardians/surrogates is in the next section. We use the National Institute of Mental Health definition of family as a "network of mutual commitment."[27]

Table 4. Multi-site cancer patient population ages 14 up to 20 years, number of dyads

Clinic Sites	Estimated Patient Population	Estimated Randomized Dyads
St. Jude Children's	317	65
Akron Children's	203	65
**TOTAL**	**520**	**130**

The current R01 proposal is based upon a previous pilot study approved by the Children's National IRB (See **Appendix B** for Information Sheet, Consent and Assent documents). We plan to modify this pilot study into a long-term protocol, in addition to developing a multi-center protocol to facilitate the investigators and local IRBs in the review process. The patient eligibility criteria have been developed with careful consideration to ensure the safety of the study participants.

Participant Eligibility Criteria

AYA Inclusion Criteria:

- Ever diagnosed with cancer;
- Knows his or her cancer status;
- Ages of 14 up to 20 years;
- Ability to speak English;
- Absence of active homicidality or suicidality, determined at baseline via screening by trained research assistant using the Diagnostic Interview Schedule for Children-IV (DISC-IV);[144]
- Absence of depression in the severe range or (>28) on Beck Depression Inventory II or score of one on item 9 assessing suicidality.** [116]
- No evidence of developmental delay*;
- Not in foster care
- Consent from the legal guardian for adolescents aged 14-17;
- Consent from a surrogate for adolescents aged 18-20;
- Assent from adolescent aged 14-17;
- Consent from adolescent aged 18-20;

* Exception: In our clinical experience, some adolescent patients with developmental delays manage their own medical care and are functionally independent, operationally defined as able: (1) to schedule and keep their medical appointments, (2) to manage their medication and know the names of their medicines, and (3) to travel to and from the clinic unaccompanied. Many of our patients come from impoverished environments. Their IQ score is not a valid measure of their intelligence, judgment or competence to make a decision about their EOL care. Cultural bias in IQ testing can also lower IQ scores. For these reasons, we propose including adolescents who have a known IQ between 60-69 who are functionally independent regarding their health care, using the three criteria listed above.

** Having a life-threatening illness or a child with a life-threatening illness can be depressing. Those participants who reach the cut off on the BDI-II will receive a structured diagnostic interview to determine if they meet DSM V criteria for moderate to severe Major Depressive Disorder. If a person does, he/she will be referred for treatment. With their consent/assent, families will be contacted monthly to determine if he/she has followed-through with referrals and received treatment. Study staff will review the potential participant's endorsement of item 9 on the BDI-II regarding suicide. Those who endorse option 2 or 3 on item 9 will be referred for further assessment. See **Adequacy of Protection Against Risks** for more details.

Inclusion Criteria for Legal Guardians of Adolescents Age 14-17:

- Legal guardian of assenting adolescent participant;

- Knows cancer status of adolescent;
- Adolescent willingness to discuss problems related to cancer with them;
- Age 18 or older;
- Ability to speak English;
- Absence of active homicidality, suicidality, or psychosis determined at baseline screening by trained research assistant (RA) as assessed by questions from the DISC-IV;[144]
- Absence of depression in the severe range or (>28) on Beck Depression Inventory or score of one on item 9 assessing suicidality. [116]**
- Consent to participate; Consent for his/her adolescent to participate;

<u>Inclusion Criteria for Surrogates of AYAs Age 18-20:</u>
- Selected by adolescent aged 18 to 20;
- Knows cancer status of adolescent;
- Age 18 or older;
- Ability to speak English;
- Willingness to discuss problems related to cancer and EOL;
- Absence of active homicidality, suicidality, or psychosis determined at baseline screening by trained RA as assessed by questions from the DISC-IV;[144]
- Absence of depression in the moderate to severe range or (>28) on Beck Depression Inventory or score of one on item 9 assessing suicidality. [116]**
- Consent to participate;

Persons who are significantly depressed are less likely to be competent to participate in shared decision-making about their own or loved ones' EOL care. If, with treatment, this changes, the dyad will be included and randomized. Individuals who are moderately-to-severely depressed, actively suicidal or homicidal at the time of screening, will also be excluded. If, with treatment, this changes, the dyad will be included and randomized. Dyads in which one member is ready to participate and the other is not will be excluded. If later, both are ready to participate, the dyad will be included. "Not ready" participants who assent/consent to be followed will be contacted regularly. They will be randomly assigned, if and when, the dyad becomes ready to participate.

Because of its sensitive nature and the use of legal documents, youth who are in foster care will not be recruited to participate in this study. The requirement for understanding of spoken English is necessary for an appropriate level of participation and because they will be signing a legal document in Session 3. Study personnel are experienced in providing accommodations for illiterate parents and family members, and these accommodations will be provided, including reading questionnaires to all participants.

Sources of Research Materials

Data collection will be confidential unless specifically required to be disclosed by state or federal law. Participants are assigned unique identification numbers used on all case report forms. Only sites will have information linking subject's personally identifiable information to the study ID number. Sites maintain all study files and documentation in a secure area where access is limited to study personnel. Participants will not be personally identified in any publication that may come from this project.

There will be a Data Sharing Plan implemented at the end of the trial. See **Resource Sharing Plan**.

Research data will be gathered either by direct entry into a web-based database via protocol specific electronic forms, or, if the internet is not available, onto paper forms and later entered into the web-based database by research assistants. All questionnaires are gathered from both AYAs and families using direct data entry into REDCap (see Methods), and by medical record review. The self-report questionnaires will be administered orally by the RA-Assessor who will record the responses by direct data entry into the web-based Children's national data base, or if using paper forms by filling in bubbles with a number two pencil or a black ink pen onto paper questionnaires that have been created for each measure.

In addition, if family consent is provided, the Session 2 of the FACE-TC intervention will be recorded by camcorder or audiotape (family preference) and reviewed by the PI, Dr. Lyon and Co-I, Ms. Briggs, for

implementation of quality control with regard to fidelity to the protocol, avoiding contamination of the control condition and monitoring for safety. Families who decline to be audiotaped or recorded by camcorder will still be included in the study, but their data will not be available for fidelity check.

This proposed project has chosen to utilize face-to-face data collection over Audio computer-assisted self-interviews (ACSI). The rationale for this choice of data collection was based on sensitivity of the interview content, previous patient perception, and the potential for an improved risk assessment.

a. Topic sensitivity: Due to the sensitivity of the topics discussed during the study interviews, we felt face-to-face conduct was preferable to ensure social respect to the study participants. In addition, our Community Advisory Boards also recommended utilization of the face-to-face format based on the sensitivity of the discussion.

b. Patient perception: Participants from the pilot study relayed a positive experience from the face-to-face assessments and interviews and viewed it as a caring experience, versus an impersonal means of data collection.

c. Improved risk assessment: This technique will provide an additional way to protect the patients by providing study staff with the opportunity to directly observe emotional distress, or any additional risks, by using our standardized procedures described under **Adequacy of Protection Against Risks.** We will defer the study visit if privacy cannot be maintained, unless participants agree to the study visit, despite the presence of other patients/family members. We will code for this in our data.

Potential Risks to Subjects

Potential risks associated with the study interviews include:

1) Emotional distress or family conflict related to discussion the death or dying of one's own self or child.

2) Disagreements may emerge regarding palliative care and EOL decision-making with regard to EOL treatment preferences. A primary purpose of this study is to contain the strong feelings conversations about EOL care may elicit and to use transactional stress and coping theory to problem solve any differences through the FACE-TC intervention. In the experimental group, the likelihood that care is in accord with patient's preferences is higher than in the "usual care" condition and this is one of the reasons for the conduct of this study.

3) Conflict may emerge regarding the selection of a surrogate decision-maker (Health Care Power of Attorney). The training program includes ways to guide the selection of the surrogate decision-maker at the time of enrollment, if the adolescent is 18 years of age or older.

4) A risk common to all research studies is breach of patient confidentiality.

5) There are no physical risks associated with the study procedures.

2.Adequacy of Protection Against Risks

A. Informed Consent Process

The proposed research will be reviewed and approved by the participating site's local IRB. Each site's principal investigator/designee will obtain written informed consent using locally approved consent form documents. As this is a multi-site protocol, the Clinical Research Coordinator at Children's National will ensure the template consent form is used by each site. Although each site's IRB may have differing required elements, the clinical coordinator will ensure that the essential required elements for consent are contained within each form, and that all forms are HIPAA compliant. The Clinical Research Coordinator will ensure that annual review and re-approval of active projects has been accomplished in a timely manner and complies with regulations from the Department of Health and Human Services, GCP and HIPAA guidelines.

Participants will be recruited by the investigators and other personnel at the participating sites. Families will be presented with the Information Sheet when first approached after initial screening for eligibility (e.g. age, diagnosis). If interested in participating, informed consent will be processed at the beginning of the secondary screening visit by the RA, allowed to read over it, and then any questions they have concerning the clinical trial will be addressed. Similarly, the AYA's assent/consent to participate in the research project will be obtained. Each participant will receive a copy of his/her signed informed consent, and an additional copy will be filed in his/her medical records.

Protection Against Risks

Dr. Lyon will be primarily responsible for protection against risks. However, protection against risks is the responsibility of all members of our research team.

Our patient sample is treatment and disease experienced. Many have participated in research studies throughout their life. They are quick to know if they want to do a study or not. Adolescents with cancer who are seriously ill and/or are at the end of life may be at greater risk of emotional distress. In conferring with our Co-I, **Dr. Hinds**, who has conducted many studies with children dying of cancer, we will implement the following as overall guidelines:

1. "Commitment to no surprises"
    a. Hinds's research shows there is decreased likelihood of a feared distress reaction if patients and families know exactly what is going to be done during the study, so first the family will be shown the questionnaires, survey, Respecting Choices Interview; Questions or Five Wishes, and then asked, if it is okay to continue;
    b. Next we will show the questionnaires etc. to the adolescent and ask the adolescent if he/she would like to continue; and
    c. The RA will state, "You could be bothered by some of these questions. You have a right to say pass. You are in complete control and you can stop anytime you want."

2. If something did upset a participant, after supporting the family, the RA will ask if there is a staff person they feel really good about that they would like to meet with and we will contact that staff person to support the participant.

In Dr. Hinds' years of experience conducting research with children with cancer at the end of life, they never had a distress situation. In Dr. Hinds' experience, children know what is right for them and with no pressure from the RA or family member will be free to say, "This study is for me or not for me." Out of 200 participants in Dr. Hinds' study they received only 2 negative comments: "I thought I wouldn't miss my son so much if I did the study, but I still do." and "This was an important study, but the timing stunk." At the end of each study visits we will ask, "What was good about this study visit?" "What was bad about this study visit?" "Is there anything else you would like us to know?" These qualitative comments will be recorded by the RA-Assessor.

Patients requiring ICU admission are very ill and will not be "bothered" by activities unrelated to their crisis. Each study site has a system in place to notify research staff of the death of a patient within 24 hours. This will prevent the risk that a family will be contacted about a study visit by the RA who does not know that the patient died.

Serious Adverse Events: Monitoring will occur on a daily basis to review any serious adverse events.
For the purposes of this study a serious adverse event will be defined as:

Definition of Adverse Events (AE)

An AE occurs when:

- In response to "What was bad about this study?"
    - "It was too much to handle." OR
    - "It was harmful." OR

Any similar negative statement reported freely by the adolescent or caregiver/surrogate or upon inquiry by the RA. If one of these responses is given, the RA will probe, e.g., "You said that Session 1 (2, or 3) was too much to handle (or harmful or both). Tell me more." The response of the participant will be recorded verbatim by the RA.

AND

- In response to "What was good about this study?" Stating any of the below. Again the RA will probe for more information by saying, "Tell me more."
    - "It was not useful."
    - "It was not helpful."
    - "I felt unsatisfied."
    - "Nothing."
    - "It was not worthwhile."

Definition of Serious Adverse Events (SAE)
Monitoring will occur on a daily basis to review any serious adverse events. For the purposes of this study a serious adverse event will be defined as an emotional breakdown requiring hospitalization or inpatient behavioral health services for emotional distress very likely/certainly related to the study intervention.

Any serious adverse event occurring during the protocol conduct will be reported and reviewed by the PI, Dr. Lyon. Any adverse event will be monitored to ensure the adverse event was actually caused by the intervention, and not some coincidental issue. These events will be collected and reported in compliance with the local IRB guidelines. The PI and local IRB must be notified within 24 hours of the reported event. The PI and local IRB must be notified not more than 5 days after becoming aware of the event. We will discontinue the study until a corrective action plan is in place. Training will continue throughout the course of the study to minimize the likelihood of such an SAE.

Confidentiality: Patient confidentiality will be appropriately protected in the process of data collection and storage at the sites. The protocol operational team will minimize the likelihood of breach of confidentiality by minimizing collected Public Health Information (PHI), using randomly generated identification numbers. Questionnaires will contain only a study specific ID number and no other identifying information. Patients will be identified only by study ID number and visit type and date. All documents and information pertaining to the study will be kept confidential in accordance with all applicable federal, state, and local laws and regulations. Research data and records collected on participants in connection with this project will be held in confidence unless specifically required to be disclosed by state or federal law. Children will not be personally identified in any publication of the data from this project.

Not all patients will be in private rooms; however, end-of-life cases usually are (especially to accommodate family members who may want to be continually present). Care will be taken to assure privacy during any bedside interviews. Moving from the hospital room to a private area will be done if possible. Attempts will be made to conduct the study when participant is alone. The same procedures will be used for home visits.

Emergency Intervention, non-protocol related: Each study site is equipped with its own protocol for emergency interventions (i.e., to address signs of acute mental illness, sexual abuse, physical abuse, or neglect) that they will implement in such circumstances. In cases of child abuse or neglect, a report will be made to Child Protective Services or the appropriate agency at each site. In every case, the surrogate will keep a copy of the surrogate and adolescent consent and assent forms, which contains emergency contact information for Dr. Lyon, and other members of the multidisciplinary team. Another copy will be stored in research offices that are triple-locked.

Emergency Intervention, protocol related: We are fortunate that in each of the settings from which participants will be recruited nurses, psychiatrists, psychologists, social workers and case managers are part of the health care team and therefore available to our research participants. Each study site has an on-call mental health specialist available 24 hours a day and an on site Emergency Room or Emergency Department. Study personnel will have clinical experience and will be trained in active listening and in issues related to cancer and death and dying. Thus, the study staff and the hospital-based setting are well equipped to address participants' emotional distress. In addition to the specific study procedures to be described below, appropriate referrals will be made to participants for additional help, such as counseling, assistance with insurance coverage, housing, and substance abuse treatment.

Patients in pain or fatigued: We will stress the absolute voluntary nature of the study and offer to do the intervention or assessment on the "installment plan" breaking up the assessments to 15 minute increments if need be and/or the intervention. Patients/families will be offered the option of discontinuing and resuming at a later time within the "protocol window" for the study visit. This leaves the option to the patient and family to decide, respecting their choice. RAs are trained to be sensitive to the needs of patients and their families and will discontinue the study visit for at a participant's request.

Participants in emotional distress: There are three study phases where emotional risks will be assessed or directly observed: 1) screening for eligibility; 2) during the 3 session intervention; and 3) during the 3, 6, 12 and 18 month post-intervention assessments.

1. *Screening.* Following the consent/assent process, adolescents and guardians/surrogates will complete a brief screener. Participants will be informed that should the screening reveal any evidence of a significant mental health issue, they will be referred to a mental health professional at the clinic/hospital or in the community for further assessment.

Immediately following completion of all screening questionnaires, the RA-Assessor will review the potential participant's responses to identify active psychosis or homicidality (assessed by interview using the relevant DISC-R structured questions), suicidality (moderate to severe levels of depressive symptoms, as evidenced by scoring above the cut off of 28 on the BDI-II, or endorsement of response level 2 or 3 on item 9 assessing suicidal ideation), or impaired mental status. Adolescent or surrogate endorsement of current suicidality, homicidality, or active psychosis will activate the following procedures at each site:

- The research assistant (RA) will contact the site Co-I to help determine if there is a clear or immediate danger. All of the site Co-Is are experienced in these kinds of assessments for dangerousness, or;
- If the RA cannot reach the site Co-I, the RA will contact/page the psychiatrist/social worker on call, who depending on the site, will either come to the research office to assess whether or not there is a clear and immediate danger to self or others or conduct the assessment at the emergency department;
- If the on call psychiatrist cannot be reached, the RA will escort the participant to the emergency department;
- In each instance, the RA will remain with the participant until the appropriate health care provider takes responsibility for the assessment and care of the participant.

Participants assessed as not in clear or immediate danger will be referred for counseling which is available at each of the site clinics or in the community. Co-Is at each site will refer the participant to the primary health care provider, if screening has indicators of impaired mental status. We will also inform participants that they may be eligible to participate at a future date, if symptoms impairing competency in decision-making resolve. In our pilot study one potential surrogate, the mother of the adolescent participant, had alcohol-related dementia. The mother sat in with the father and the son for the study, but the father was the surrogate. Every effort is made to be as inclusive as possible of family members important to the adolescent and family.

2. *Intervention.* Although we had no adverse events during our FACE-TC pilot study, we appreciate that despite the structure of the FACE-TC intervention, which was designed to contain strong emotions in a supportive, caring and respectful way, some participants may become aware of painful feelings of guilt, anger or sadness. If an AYA or family member is overwhelmed by strong feelings, the RA-interventionist who is clinically experienced and trained in active listening and affect regulation, will provide support. After support is provided the RA will ask if the family would like to continue, or take a break, or reschedule for another day. We will not remind the family at this time that they can discontinue the study, as they may experience this as abandonment or punishment for expressing their feelings. However, participants will have been told during enrollment that they may withdraw from the study at any time and will be reminded of this when confirming the next scheduled appointment.

If the AYA or family member continues to be distressed, the RA will ask if there is a staff person they feel really good about that they would like to meet with and we will contact that staff person to support the participant. If yes, the RA will stay with the family until the staff person arrives or accompany the family to the staff person's office and wait with the participant until the participant is seen.

If the AYA or family member, during the course of the intervention expresses suicidal, homicidal or psychotic thoughts the procedures for managing this event will be followed as described under "1. Screening."

3. *Assessment at 3, 6, 12 and 18 months post intervention.* The same procedures will be followed as described during the screening process.

To further protect against risks, we will continue with the following protections, which we put in place for our American Cancer Society pilot:

1) We will hire staff with experience, maturity, good basic communication skills and good interpersonal skills;

2) RAs hired to be RA-interventionists who do not meet certification criteria for the Next Steps: Respecting Choices Interview will not be allowed to interact with participants;
3) We will provide ongoing intensive and comprehensive training. For example, in addition to what was described earlier, RA-interventionists participate in two days of multiple role plays of possible adverse events that could occur during the Respecting Choices Interview. Scenarios will be given which were known to have ever come to the attention of the ethics board at Children's National, so that we will be as prepared as possible for any eventuality;
4) Site supervisors will provide weekly supervision of the RA-Interventionist and RA-Assessor;
5) The research team at each site will meet weekly with the site investigator to provide support to the research team and to address any emotional reactions or concerns they (RAs) have about a new family under consideration for recruitment and enrollment or issues that arose in the course of an intervention session;
6) We have a chaplain and an ethicist at each site available for referral to discuss spiritual struggles or to help process conflicts about treatment preferences;
7) Procedures are clearly delineated about what the RAs should do if a participant should become suicidal or homicidal or psychotic during the intervention sessions or during any of the assessments; and
8) The site Co-Is are available by pager or cell phone and this information is always available in our consent and assent forms. All research participants have a copy of the assent/consent form, which will contain appropriate Investigator pager numbers.

In addition to the procedures already in place, if they should prove to be insufficient, we also have an additional co-investigator at the Children's National site, Dr. Hinds, who is an expert in safety issues in conducting research with children dying of cancer. Dr. Hinds and Dr. Lyon share an office suite at Children's National and have easy access to one another for consultations. Dr. Lyon, the site Co-Is and the Safety Monitoring Committee, as well as the study staff, will be responsible for ensuring that these procedures are followed and documented in the process notes and that the safety of participants is protected.

Although this study will be examining spirituality/religious experiences and beliefs, the FACE-TC study is not a religious or spiritual intervention. We are including everyone regardless of religious affiliation. Instructions for responding to assessment measures include a statement that "Not all of these questions may apply to you. Please answer as best you can." Data on religious affiliation or no religious affiliation will be collected. If an adolescent gives no response, because it does not apply to him/her, the data will be coded as missing data.

We will verify with the site Co-Investigators, using the Co-Is' judgment, that the patient is not too impaired by pain medication/alertness level to participate in the study visit, before proceeding with a study visit for a patient who is in the hospital or Hospice care. The location of the patient at the study visit (outpatient, inpatient, hospital room or Hospice care) and competency to participate will be coded for each study visit. If a patient should die after completing the FACE-TC intervention, we will check the medical record to see if the adolescent's wishes were honored and if the person the adolescent selected (if over the age of 17) was the decision maker. This data will be used for exploratory purposes.

Respondent Burden, defined as the subjective phenomenon that describes the perception by the subject of the psychological, physical, and/or economic hardships associated with participation in this research study,[145] has been reduced in the current proposal 1) by reducing the number of questionnaires; 2) by periodically revisiting assent/consent of the patients involved in the study; and 3) by ensuring that patients will feel free to decline research participation by eliminating the health care provider (HCP) from being involved directly in recruitment of participants; and 4) by training recruiters not to suggest during the recruitment process that their HCP recommended them to the study. In our American Cancer Society pilot study participants felt cared about and satisfied with the benefits of participation (that adolescents wishes were known for EOL care).[28]

Recruitment and Retention Strategies
All sites have a history of success in recruiting adolescents with cancer and families for participation in research studies. Currently, all sites are running clinical trials. We are especially sensitive to the distress of families and adolescents living with cancer and to recruitment during the EOL.[146,147] To prevent any perception of coercion or desire to please the primary Health Care Provider (HCP), the HCP will not be involved in the recruitment of participants. We will generate a list of potentially eligible participants and follow the procedures described in **Approach.**

Attrition. See **Approach** for data on attrition at study sites. In the PI's judgment, our study sites have such low attrition because patients form strong attachment bonds with the clinical and research staff, a "positive institutional transference" (David Reiss, M.D. personal communication, former director of the Family Research Center at George Washington University). Families trust us and have known us for years. Thus, we conservatively estimate that approximately 30% of families will withdraw (25% due to death, 10% dropout) by the 18-month post-intervention assessment. We estimate that there will be approximately 91 dyads (n=182 participants) with full data from all sites at 18-month follow-up from the original sample of 130 randomized dyads (n=260 participants).

The process of identifying possible families for participation is described in **Approach**, but repeated here for convenience of the reader. A master list of potentially eligible participants by age is generated by the Research Nurse Coordinator. All patients who meet exclusion criteria (e.g., being developmentally delayed), are then eliminated by the case managers or Clinical Research Assistant (CRA) at each study site. A list of potentially eligible participants is then generated to approach for screening. The research assistants attend weekly multidisciplinary team clinical care meetings with Health Care Providers, psychologists, social workers, dieticians and case managers who review patients who have come into the clinic the previous week. Using their master list of potentially eligible participants, patients who presented to the clinic in the previous week and who are thought to be eligible are identified for recruitment in the group meeting. The study RA then contacts the adolescent or guardian with the help of case managers or CRAs. The RA/CRA, not the HCP, contacts the family to inform them about the study. Families may also be contacted at the time they present for a medical visit or by telephone. This process may be amended/adapted to the specific processes at each study site, as required. All potential eligible patients will be contacted until recruitment goals are reached. However, in our experience, those who have recently presented for care are easier to contact initially (e.g., have up to date phone numbers), which is why we start there.

In the REACH Study, funded through ATN/NICHD/NIH, adolescents in this multi-site study rank ordered influences on recruitment and retention. For *recruitment*, the top 5 influences were: quality care, caring staff, health education, honor privacy and altruism (tie). For *retention* in this 5-year study, the top five influences were: quality care, caring staff, health education, honor privacy, and altruism (3-way tie). Ranking 9th, 10th, and 11th were compensation, transportation, snack/food meal.

Part of our success in recruitment and retention is due to our staff members who are trained to be sensitive to the specific needs of adolescents, particularly privacy and their rights as a research participant. At the time of recruitment, adolescents complete a "Contact Information Sheet." It asks: If I call your home, what kind of message is o.k. to leave? What is your preferred way for the research staff to contact you? Is it o.k. to send you something in the mail? What type of envelope do you prefer? One with a Hospital logo or a blank envelope? If you have cancer, who at home knows your diagnosis? Is there anything else you would like us to know when contacting you? This process establishes rapport and builds trust.

Appointments are scheduled to accommodate the needs of patients. RA availability will average 12 hours a day, and flexibility in availability to families is a key qualification for the job. RAs are selected, in part, based on the flexibility of their schedules, so that appointments can be made at times most convenient for families, including early mornings, evenings and Saturdays. The Information Sheet (**Appendix B**) scripts the way in which the study will be described to the patient. During training, how to approach families is role played. If the AYA is interested, we will ask permission to contact his or her legal guardian. If the AYA is 18 years of age or older, the researcher will help him or her select a surrogate, as described below. A member of the research team will then ask the adolescent for permission to contact the surrogate about participation in the study. Using the Information Sheet, the study will then be explained to the legal guardian or surrogate by telephone or in person. An appointment will then be scheduled to describe the study in detail and to screen and enroll (consent/assent) participants into the study. This process usually takes 35-60 minutes, as we always take the time to assess readiness and potential obstacles to completion of the study. What will be required of study participants will be described. The RA will also describe compensation for time, transportation, and childcare for participation in the data collection, as well as meal vouchers for use while at the study site. Once recruited and enrolled, AYAs and legal guardians/surrogates will receive reminder calls, postcards, birthday cards, and appropriate holiday cards from an RA. Three sources of contact information will be obtained from each dyad.

The assent/consent process describes the study in great detail. Our research experience is that the assent/consent process is the best time to assess readiness to participate, enhance motivation, and eliminate individual or family barriers to participation. In a minority of cases, there will be two legal guardians. These choices create a number of dilemmas, addressed below.

Study Procedures for the Selection of Legal Guardians/Surrogates for AYAs
There are important concerns about basing the selection of a family member to participate on the AYA's preference. After consultation with Ms. Briggs about the selection of surrogates in their studies and after consultation with the Family Ties Project, which has helped HIV+ parents do contingency planning in Washington DC since 1980, we have developed the following procedures for the selection of surrogates. For adolescents aged 14-17, only legal guardians will be allowed to participate because they are legally responsible. At the time of recruitment, but before assent and consent to participate in the study, the adolescent will be asked permission to contact his/her legal guardian(s) about participation in the study. If the legal guardian is seriously ill and the adolescent and legal guardian agree, the legal guardian and adolescent during the assent/consent process may select another adult to act "in the place of" the ill guardian with the understanding that the legal guardian's consent and signature will be needed on all documents.

We understand that the process of decision-making might be changed by having either one guardian or both present, and may also vary as a function of the gender of the guardian. We are concerned about asking an adolescent to choose the legal guardian he/she would prefer, as it may raise feelings of divided loyalty. Also, the guardian the patient would feel most comfortable with discussing personal issues may not be the same person who has the power to make important decisions in the family. For these reasons, we invite both guardians, as this is consistent with clinical practice. Nevertheless, in our pilot study, the majority of the surrogates were biological mothers.

The nationwide study by Emanuel and colleagues [148] demonstrated that women are the predominant caregivers providing assistance in the care of terminally ill patients. In Session 3 the intervention dyads will choose a first, second and third surrogate, as part of the completion of the Five Wishes. We have decided for the current R01 application to use the data from the person selected as the first surrogate in our data analysis. This person, will by definition, always be present during this session.

FAYAs aged 18-20 will be asked during recruitment to choose a surrogate, using the guidelines below. Respecting Choices® recommends four criteria to consider in choosing a health care surrogate. In addition to a brochure about surrogate decision-makers, we propose asking the adolescent aged 18-20 to consider these criteria in choosing a surrogate:

1. Is the person willing to be a surrogate? Sometimes even the most trusting and loving people would find this role very difficult.
2. Do you trust the person to know your views and be willing to talk with you?
3. Is the person able to follow through and honor your wishes, even if they might not agree with your choices?
4. Can the person make decisions under sometimes stressful and difficult situations?

The selection of a surrogate decision-maker, role/responsibilities and preparation of a surrogate are all content that is covered in the 2-day face-to-face Research Assistant Training.

Resolving Disagreements
We are taking a very cautious approach and want to note that the issues to be addressed next, did not arise during our American Cancer Society pilot study. Nevertheless, we recognize that given the potentially charged nature of the study. In our pilot studies the intervention proved sufficient to resolve differences about EOL preferences and there were no adverse events. Nevertheless, a referral to the site chaplain or ethicist or mental health specialist will occur in the following instances: (1) To identify a surrogate decision-maker, if there is conflict in the family about the adolescent's choice; (2) To provide support and to continue to process feelings identified during the course of the intervention, if conflicts in treatment preferences for EOL care emerge during the intervention. Processes, such as labeling feelings and concerns, as well as finding solutions to the identified problem, will be facilitated. The RAs focus on what the family members have in common,

which usually is what is in the adolescent's best interest. An atmosphere of respect and active listening is created for the adolescent's perspective.

If there is conflict between the wishes of the adolescent for palliative care and the wishes of the legal guardian(s) that cannot be resolved during the course of the intervention, the Co-Is will consult with their site ethicist. Families may also be referred to their site Ethics Board. In addition, Tomas Silber, MD, MAAS, Director of the Office of Ethics at Children's National and former Chair of the Committee on Ethics of the American Academy of Pediatrics is available for consultation with the PI in the study. If there is a spiritual or religious struggle, adolescents and family members expressing a desire for spiritual counseling will be referred to an appropriate spiritual counselor at the site. The PI will also consult with Kathleen Ennis-Durstine, MDiv, Senior Chaplain and Manager of Spiritual and Pastoral Care, APC at Children's National, as needed.

Informed Assent/Consent (Appendix B) of Patients and Surrogates

Only study team members trained to secure assent/consent will approach participants about the study. Written consent forms will be completed and all consent procedures will be documented according to the standard hospital procedures. All data will be kept confidential and stored in a locked file inside a locked office. Refusals will be documented in the research records and examined for any possible patterns. All participants who meet eligibility criteria regardless of gender or minority status are fully eligible to participate in this study. If they choose to enroll, the patient's and guardian(s)'s written assent and consent will be obtained by study interview staff at screening. Adolescents aged 18 up to age 20 only need to provide consent. Patients and the legal guardian(s)/surrogate(s) may decline or withdraw from the study without penalty at any time.

An appointment will be made for assent/consent and screening. All participants will be asked to complete a brief demographic data sheet to ensure that there are not significant differences between acceptors and decliners. These sheets will be assigned a unique identifier number. Assent/consent at this time will be for data collection for adolescent and guardian/surrogate at five points in time: baseline assessment; 3-month post-intervention, 6-month post-intervention, 12-month post-intervention and 18-month post-intervention assessment.

Assent/consent will also include a release to give their primary HCP a copy of the Statement of Treatment Preferences and the Five Wishes, advance directive, and to alert their HCP if the mental status exam suggests the need for further assessment. A copy of the Statement of Treatment Preferences, Five Wishes (as attachments) and a summary statement of the respecting choices conversations about goals of care in a HIPPA compliant email will be given to the primary health care providers for their records. No other data will be shared with the health care provider.

Assent will be obtained for all youth under the age of 18. Parental or legal guardian consent to participate (for both the youth and themselves) will be obtained for all youth under the age of 18 unless they are legally emancipated minors. AYAs aged 18-20 and their surrogate will consent to participate. Participants will be assessed to determine readiness to participate, to identify obstacles to participation, and to enhance motivation. They will be given ample time to answer questions. This process is expected to take 35 to 60 minutes.

The consent and assent forms describe the nature of the screening phase, right to refuse to answer any questions and to withdraw from the screening process at any time without penalty, methods in place to protect confidentiality, and limits of confidentiality (e.g., suicidal/homicidal intent). The consent form describes the nature of participation, rights of refusal to answer any question and to withdraw at any time without penalty, methods in place to protect confidentiality, and limits of confidentiality (e.g., child abuse/neglect, suicidal/homicidal intent).

Eligible adolescents and families will then be scheduled to complete the baseline data collection session. This time delay gives the family time to fully consider the commitment to participate. At the completion of the baseline assessment, the Clinical Research Coordinator at Children's National will scan their questionnaires into a computer within two days, which will randomize the dyad. Dyads randomized to the intervention will then be scheduled for Sessions 1-3. The RA-Interventionist will receive an email from the Children's National Clinical Research Coordinator, informing him/her of the randomization. The RA-Assessor will not be informed

of randomization, to maintain the blindness. The RA-Interventionist will then contact the family to confirm the first intervention session, to explain the program in more detail and to answer any questions. The RA-Assessor will administer all follow-up assessments at 3, 6, 12 and 18 months post-intervention (approximately 4 weeks after the baseline visit).

Randomization will be triggered when baseline assessments are scanned into the computer by the Clinical Research Coordinator. Employing an intent-to-treat paradigm, dyads will be randomly assigned to FACE-TC vs. TAU CONTROL, blocked by study site. Participants will be blind to assignment until Session 1. An RA-Assessor, not the interviewer, administers post-session questionnaires orally, recording responses onto standardized questionnaires for later entry into the web-based database or directly entered into the computer laptop and checked for validity immediately following data entry.

Participant Reimbursement

There will be no reimbursement for consent/assent meeting, screening for eligibility or completion of the Demographic Data Sheet.

After a review of the literature,[149-155] a review of the policies of the Adolescent Trials Network (ATN) which conducts randomized clinical trials with adolescents who are HIV+, and our experience with our ACS pilot study, we have decided to provide monetary compensation to the adolescent and legal guardian(s)/surrogate for their time, effort, and the inconvenience our research entails. We chose not to use savings bonds, gift cards or movie tickets for four reasons: 1) adolescents with cancer in the pilot study preferred reimbursement in the form of cash; 2) many of our adolescents live in inner cities where access to shopping centers and movie theatres are difficult without a car, so vouchers might not be used; 3) in our pilot, we have found that monetary reimbursement (given immediately in cash) was preferred by surrogates and adolescents (compared to gift certificates) and 4) research shows that gift cards are often not used and expire. For these reasons we decided to use a monetary reimbursement. Unless other arrangements have been made, reimbursement for time and inconvenience will be immediately upon completion of each intervention/assessment session. We appreciate that there is a risk that the adolescent will have the reimbursement taken from them by their surrogate. However, this did not occur during our pilot and we have judged this to be a risk worth taking for the aforementioned reasons. In our judgment and that of medical ethicists monetary reimbursement for time and inconvenience is a respectful way to treat our adolescent and adult research participants.[156]

To ensure that the payment does not constitute an undue inducement, particularly for our more vulnerable adolescents with cancer who may be medically unstable, the reimbursement for time and inconvenience described below is lower than the norm for the Adolescent Trials Network (ATN) youth who are HIV+ and for the study sites generally. Adolescents and legal guardians or surrogates will receive $25 each for the Baseline assessment, which will require approximately 60 minutes. They will also receive $25 each for participation in the three 60 minute intervention sessions. The three sessions will be scheduled one week apart, ideally on the same day of the week at the same time, as families tended to remember this routine. Participants will receive $25 each for completing the 3- and 6-month post-intervention follow-up. Participants will receive $30 for the 12-month assessment and $30 for the 18-month assessment of approximately 60 minutes. Participants will be reimbursed transportation fees for travel to and from the clinic. Parking stickers will be available. Money to cover babysitting expenses will be provided. Meal tickets to the cafeteria will be provided at the time of each visit. The rates of compensation for travel and meals are consistent with the norms for the study sites. Participants will be offered a snack break to prevent participant fatigue.

The Role of the Physician or Primary HCP/Follow-up Interview Activities

Physicians are not conducting the FACE-TC intervention. If patients and/or their legal guardians have unanswered questions regarding their medical condition, potential complications, and benefits/burdens of life-sustaining treatment choices, they will be referred back to their physician or appropriate HCP to seek clarification. A postcard is given to the adolescent with their questions on it, as a cue to remind them to ask during their next medical visit. In our pilot focus groups, adolescents found this empowering. Patients will be assisted in developing specific questions they have for their physician and be encouraged to discuss treatment decisions with other professionals, such as a religious advisor. Any treatment decisions made by the legal guardian or adolescent aged 18 or older will be documented in the medical record as evidence of their wishes

in accordance with the standard practice at each site for documenting advance directives. No data from the primary HCP will be collected during this study.

Protection Against Breaches of Confidentiality
Participants' data will be kept confidential. Participants will be told that they may choose not to answer any given question. No confidential information concerning patients' charts will be released to us, until patients/clients provide explicit permission by completing a signed release. At the time of assent/consent, participants will sign a release of information to share a copy of their Statement of Treatment Preferences and the advance directive, The Five Wishes© with their primary HCP. Participants will understand that this study has clinical medical purposes, as well as research purposes. They will also be informed that the completed Five Wishes© along with the Statement of Treatment Preferences is both a research document and a medical directive document and will be placed in their medical chart. With the exception of the primary HCP having a copy of the Statement of Treatment Preferences and the Five Wishes, only site research staff will have access to the participant's identity. The research documents will contain a copy of Statement of Treatment Preferences and the Five Wishes© with the patient and surrogate signatures covered over during photo copying and the unique identifier in the signature space. The original documents will be placed in the medical chart.

Consent and assent forms will describe the nature of participation, rights of refusal to answer a question and withdrawal at any time without penalty, methods in place to protect confidentiality, and limits of confidentiality (e.g., child abuse/neglect, clear and immediate danger to self or others). Participants sign a separate release for permission to audiotape or videotape/DVD or it can be embedded in the assent/consent documents depending on site practices.

A unique identifier number will be used on all paper surveys and for coding data on the computer. Data will be collected by the research staff and stored and locked in file cabinets in a locked office in a locked suite. The unique identifier and patient identity list will be stored in a different location from the data by the Principal Investigator, Dr. Lyon. Original DVDs, audiotapes or videotapes and copies will be destroyed upon completion of the study by cutting discs in half or erasing audiotapes. Some families gave us permission to use their videotapes for teaching purposes and for data dissemination. They signed appropriate site-specific releases, consistent with the hospital policy of the site. These DVDs and videos will assist with future training, education and dissemination, sensitive to the privacy issues involved.

3.Potential Benefits of the Proposed Research to Human Subjects and Others
Potential benefits of participating in the FACE-TC intervention to participants include relieving psychological, spiritual, and physical suffering, as well as maximizing quality of life. Psychological benefits include "breaking the ice" for ongoing conversations about treatment preferences as cancer progresses so that there is minimal decisional conflict and sustained congruence in treatment preferences. Families can then be assured that they had honored their child's/or partner's wishes and adolescent patients, in turn, can feel supported to the end trusting that their families were there for them. Although not the primary purpose of our study, our pilot data suggest that the experience of mastery and control in the context of family support in a caring, respectful way, supporting family decision-making concerning palliative end-of-life care, strengthens families, which may improve the quality of life of study participants. Adolescents and their families will have the opportunity to enhance their social support networks through involvement with the research staff, which may diminish feelings of isolation. Adolescents in the study may also improve their linkage to care, simply through participation in the study, including enhanced connectedness with their respective clinics, benefiting from any primary or adjunct services offered by the clinics (e.g. mental health, case management).

The proposed study has the potential to build an evidence base for structured pACP as one dimension of pPCEOL, thereby benefiting all adolescents living with a life-limiting condition. If this R01 demonstrates long-term efficacy, we plan to submit a future R01 to conduct international trials with adolescents with cancer in other countries, adapting the FACE-TC model through community based participatory research. Furthermore, these results may provide an empirical foundation and method (FACE-TC protocol) for relieving suffering and maximizing quality of life for all adolescents living with a life-limiting condition (e.g. cystic fibrosis, muscular dystrophy) where the prognosis of a life-threatening condition is known, but the timing and the certainty of

death is unknown, because of rapid advances in medicine, including the search for a cure. This approach is consistent with the call of the World Health Organization that pediatric palliative care planning begin early.

4.Importance of the Knowledge to be Gained
The major benefit of this proposed project will be to fill the gap in our knowledge about what minority adolescents and families want with respect to palliative care, consistent with the recommendations of the NIH State of the Science Conference Statement on Improving EOL Care[157] to increase the study of minorities and children who are underrepresented in EOL research. This study will also inform the current clinical, ethical and policy discussions as well as the legal issues in a variety of areas, such as the debate surrounding advocacy that the legal age for making an Advance Directive (AD), including a Do Not Resuscitate (DNR) order, be lowered to 14 years of age. Careful research in this psychologically sensitive area of pPCEOL is also consistent with IOM recommendations [34] and the NIH Roadmap intention to discourage "risk adverse" research and to encourage innovation.[158]

Our hope is that this study will provide a structured model for facilitating family decisions about EOL care, while adolescent patients are competent to share in decision-making. The structure of the FACE-TC model may help to contain the anxiety, sadness and anger that talking about death can provoke, meeting the goals of the NINR's campaign *Conversations Matter.*™,[8] Facilitating these discussions in a sensitive way in a clinical setting, as part of routine care, may improve quality of life and patient outcomes by increasing the likelihood that the patient's goals of care are honored in the last weeks of life. This intervention may empower adolescents and their families to make their own choices regarding palliative care and to communicate these choices to their primary HCP, thereby increasing their control and sense of self-efficacy. It will also provide us with knowledge of the impact of religion on treatment choices. If this R01 is funded, we plan to apply for future funding in the second year of this study to follow this valuable cohort for an additional 5 years to determine if adolescents' wishes are honored in the last few weeks of life, as is being found in more recent studies using this or similar approaches. We will be exploring this variable, the match between care received in the last weeks of life and the goals of care of the adolescents with cancer.

As with our pilot study, we will send study results for primary outcomes to study participants in the form of a newsletter. For those patients who may die while on study, the newsletter will be accompanied by a personal letter expressing sorrow for the loss of their child and appreciation for their participation and contribution to the science of ACP. This letter will be personalized and signed by the research staff known to the patient and family.

The risks or participation in this minimal risk study are reasonable in relation to the importance of the knowledge that may result from the study.

Study participants who were randomized to the control condition will be offered the intervention while the study is in Year 5 dissemination stage, if study aims are realized. The Co-Is at each of the study sites will commit to this.

5.Data and Safety Monitoring Plan
Data entry is an ongoing process so that entry is accomplished generally within a week after the data have been collected. The data manager regularly checks for out-of-range values or logical inconsistencies. A double-check system is used to assure accuracy and consistency of coding. Entered data are compared to the original data to assure accuracy. Weekly reports will be generated to show the status of all participants on study.

The project office is a locked room to which only study personnel have access. Original data are stored in locked file cabinets. Keys are tracked through a centralized system. Study personnel are trained in the ethics of protecting confidentiality through course work, in discussion with the PI, and by completing the NIH course on protecting human subjects. Study personnel are trained to ensure compliance with HIPAA regulations protecting patient health information.

To improve the infrastructure that supports clinical research at Children's National, it has developed an integrated research information system. It will include a relational database as well as tools that are designed

to significantly enhance study management and improve the completeness and quality of research data. The system will provide support for research design and protocol implementation, proceeding with enrollment, collection of protocol-driven data, and analysis of results. It will also assist with monitoring patient safety and data quality.

The study database will provide a highly structured repository to store and protect the integrity and confidentiality of study data. In addition to the study data itself, the database will house information about each protocol, including information describing each item of data collected. The stored information about each protocol will include the targeted enrollment quotas, eligibility criteria, as well as a detailed schedule of visits with information to be collected at each visit. This will enable the system to help with study management and to help improve study quality.

Finally, the database is directly accessible to standard statistical packages such as SAS, SPSS, and STATA so that interim and final analyses can be conducted without the need to transport data. Developed in Microsoft .Net technology, version 1 is under active development on the central research computer network. This system is accessible via the Internet network to investigators and their staffs. Security is provided via a password-based authentication system that governs access, including a data view that will be dictated by study role. In addition, patient confidentiality is protected by utilizing a randomly assigned ID number and by separating and closely controlling access to the one file that links that ID number to another universal ID that links to the patient's name or other identifying information. The ID number will never be directly linked to the identifying data.

Database Design, Data Management and Monitoring

The Biostatical and Study Methodology, BSM, Division at Children's National uses REDCap as one of its main databases for data collection. REDCap is an open source web application (http://project-redcap.org) developed by Vanderbilt University and made available to Children's National since it is a member of the Clinical and Translational Science Award Consortium. The underlying storage facility is MySQL, whose structure permits the linking of patient information across all tables like all relational databases. The web-based transmission of data is encrypted and fire-walled. When designing the data collection forms in REDCap the data management team will consider the NIH Common Data Elements and utilize the templates for questionnaires/surveys already set up in REDCap.

RA-Assessors at study sites will be responsible for entering data directly into the study website on a study specific laptop, and the Data Manager will train RA-Assessors from each study site by visual teleconference to competency. To ensure accuracy, the data will go through some validity checks at the time of entry (mostly range checks and date validity checks). Routine edit checks will run more complex queries. The intent in this approach is to allow sites to complete data entry promptly, and not slow them down except for very gross errors, and to query shortly thereafter should the data at the site show inconsistency. The Data Manager will monitor data to ensure timely and uniform collection. To minimize risks of disclosure of participants' personal information, Children's National, the coordinating center, and study sites will ensure management and protection of materials and information in compliance with national, state and institutional guidelines for protection of confidentiality of health-related research information. No HIPAA protected information will be put in the database.

Safety Monitoring Committee (SMC)

This study will be conductance in accordance with appropriate sections of the International Conference on Harmonization's Guidelines for Good Clinical Practice. The principal investigator, Dr. Lyon, will be responsible for monitoring and the process by which Adverse Events and Unanticipated Problems will be reported to all relevant regulatory bodies.

Dr. Lyon will compile a Safety Monitoring Committee (SMC), following the guidelines provided by NINR/NIH. Dr Anne E. Kazak, PhD, ABPP, Co-Director for Health Care Delivery Science at Nemours Biomedical Research

and Professor Emerita in Pediatrics at the University of Pennsylvania, has agreed to Chair the Safety Monitoring Committee. Two additional members will be invited who are experts on the science and ethics of research on pPCEOL for children. Dr. Kazak is an expert in the full range of issues associated with adolescents with cancer and their families (see **NIH Biosketch and Letter of Support**) with additional expertise in evidence-based assessment and intervention, specific to the goals of this PAR to build evidence for effective Palliative/End of Life Care Interventions.

The SMC will meet a minimum of once a year. The SMC will decide what data summaries it wants to see in order to adequately monitor the data and the safety of the patients in the study. The SMC will review all adverse events, for immediate assessment of any safety concerns in the study. All serious adverse events will be reported to the SMC and the local IRBs within 24 hours of event knowledge. The SMC will then notify Dr. Lyon and the Research Coordinator, who will coordinate notification to all participating site IRBs.

RESOURCES

Children's National Health System

EVALUATION AND TECHNICAL SUPPORT CAPACITY

Study data will be collected and managed using REDCap electronic data capture tools hosted at the coordinating center for this study Children's National.[88] REDCap (Research Electronic Data Capture) is a secure, web-based application designed to support data capture for research studies, providing: 1) an intuitive interface for validated data entry; 2) audit trails for tracking data manipulation and export procedures; 3) automated export procedures for seamless data downloads to common statistical packages; and 4) procedures for importing data from external sources.

ORGANIZATIONAL INFORMATION

Clinical

Children's National is the only exclusive provider of pediatric care in the metropolitan Washington, DC area and is the only freestanding children's hospital between Philadelphia, Pittsburgh, Norfolk, and Atlanta. Serving the nation's children for more than 130 years Children's National is a proven leader in the development and application of innovative new treatments for children. It is proudly ranked consistently among the best pediatric hospitals in America by the US News and World Report. Children's National's internationally recognized team of pediatric healthcare professionals annually care for more than 360,000 patients who come from throughout the region, nation and world. Children's National is a 283-bed facility with more than 13,200 inpatient admissions, 250,000 outpatient visits and over 75,000 ER visits in 2008. Serving as an advocate for all children, Children's National is the largest non-governmental provider of pediatric care in the District of Columbia, providing more than $50 million in uncompensated care.

Research

The Children's Research Institute (CRI) at Children's National continues to excel in research as one of the premier pediatric academic institutions in the country. Investigators from CRI have been highly successful in obtaining many competitive research grant awards from the national Institutes of Health. In FY13 the research work of the CRI is now supported by 79 primary NIH grants awarded to 50 investigators totaling $29,751,371. This statistic illustrates the dedication and support of the institution to advance pediatric research, which will lead to prevention, diagnosis and cures for many childhood diseases, as well as providing a medical home with preventive services for well children. In recent years Children's National has ranked among the top 10 Departments of Pediatrics/Children's Hospitals in terms of NIH funding. This funding is divided almost equally between clinical and

translational research projects. Children's National has particular strengths in multidisciplinary, team clinical and translational research especially in five areas: 1) neuroscience and behavioral medicine; 2) genetic medicine; 3) cancer and immunology; 4) clinical trials and experimental therapeutics; and 5) translational research.

Perhaps most importantly, there is a history of collaboration between researchers and clinical care providers at Children's National. In part because both research and patient care are housed together in one physical and institutional entity, program evaluation and clinical researchers have long been a component of patient care. Research and patient care are well integrated, and inform each other's development. Researchers and clinicians are colleagues and are welcoming of each other's contributions. This collaborative relationship is critically important in ensuring the success of participant recruitment and retention, and of ensuring fidelity through the completion of intervention monitoring forms. Clinicians at Children's National have a history of contributing to these efforts and feel comfortable in accepting of these tasks.

The Center for Translational Science at CRI, directed by Dr. Lisa Guay-Woodford, conducts patient-oriented translational research in pediatrics that leads to advancements in health care promotion and improves the prevention, diagnosis, and treatment of childhood diseases in hospitals, outpatient settings and our communities. The Center identifies the optimal methods for delivering preventive services and other health care to children and their families, paying special attention to disadvantaged and minority populations. Collaborative investigations with health policy experts and health services researches contribute to the overall research program. The Center has several infrastructural components: the Center for Biostatistics and Informatics, the Clinical and Translational Research Center, the DC-Baltimore Research Center on Child Health Disparities; the Multi-Study Section; the Office of Investigational Therapeutics; and the Rare Diseases Clinical Research Center. The services of the CTS patient advocate, **Dr. Tomas Silber**, will be provided to all research participants. The CTS is a collaborative center, which includes biostatisticians, nurse scientists, psychologists, physicians, and epidemiologists. The Research group meets monthly to discuss ongoing projects, collaborative opportunities, and functions as a forum for constructive research-related feedback.

Office
Research activities will take place in office facilities in the CTS. The CTS suite comprises 3,114 sq ft of newly renovated space including two conference rooms. Seventeen faculty members occupying this space are supported by three resource coordinators and three research assistants. Equipment available to staff and faculty includes a Canon Color Image Runner, an all-in-one network scanner, fax, printer and copier. Desktop computers with network capabilities and data analysis software are also available for Dr. Lyon.

Drs. Lyon, the PI, Dr. Hinds, Co-I, and Dr. Wang, Co-I & Bio-statistician, Yao Iris Cheng, Data Analyst and Data Programmer, have individual offices within a few feet of each other. Dr. Lyon's office is located at Children's National Main Hospital in CTS/CRI office suite on the 6th floor, Room 7568. Dr. Watson has an office in Nursing Research. Desktop computers are available for each staff member (5), in addition to Investigators have laptops for working at home, desks, chairs and lateral locked filing cabinets which are located in locked offices in locked suites.

Institutional Review of Informed Consent Documents
The proposed research will be reviewed and approved by the participating sites' local IRB. Each site's principal investigator/designee will obtain written informed consent using locally approved consent form documents. As this is a multi-site protocol, Dr. Lyon will ensure the template consent form is used by each site. Although each site's IRB may have differing required elements, the Clinical Research Coordinator, will ensure that the essential required elements for consent are contained

within each form, and that all forms are HIPAA compliant. The Clinical Research Coordinator will ensure that annual review and re-approval of active projects has been accomplished in a timely manner and complies with regulations from the Department of Health and Human Services and HIPAA guidelines.

Data Storage
Study data are maintained in a REDCap data base. REDCap is superior to ACCESS or Excel in its ability to produce clean datasets that are relatively ready for immediate analysis. REDCap provides

1) a stream-lined process for rapidly building a database or an online survey;
2) an intuitive interface for collecting data (with data validation) in a web-based environment; and
3) advanced features, such as branching logic, scheduling, file uploading, and calculated fields.

This system includes a relational database as well as tools that are designed to significantly enhance study management and improve the completeness and quality of research data. The system provides support for enrollment, collection of protocol-driven data, and data analysis. It also assists with monitoring patient safety and data quality, including data checks, logic checks. This enables the system to help with study management and to improve study quality. REDCap has a component to schedule study intervention and follow-up/assessment visits. In addition to error tracking, reports will be developed to provide timely and up-to-date monitoring of each facet of the study which now includes an alert system that notifies designated staff of key events needing attention (e.g., meeting benchmarks). Stored information including enrollment quotas, eligibility criteria, and a detailed schedule of visits with information to be collected at each visit will enable the system to confirm eligibility of enrollees, to track actual vs. projected recruitment, to develop online schedules of research visits, and to monitor protocol adherence. Finally, the database is directly accessible to standard statistical packages such as SAS, SPSS, and STATA so that interim and final analyses can be conducted without the need to transport data. Security is provided via a password-based authentication system that governs access, including a data view that is dictated by study role. Patient confidentiality is protected by utilizing a de-identified research ID number and a firewall. Data will be stored on the hard drive of the Children's National Biostatistics and Informatics Unit. Experienced personnel, who are highly skilled, maintain our computer database system and files.

Original hard copy data will be stored at the primary site in locked filing cabinets in secure rooms to which only study personnel have access. Paper questionnaires, administered when there is no internet access to the Web data base for entry, will be scanned into a study designated drive on a secure computer. After verification of data entry accuracy, the paper questionnaires will be shredded. Each site will follow local procedures for warehousing records, including maintaining records in locked files.

DVDs collected for maintaining fidelity to the protocol will be stored at Children's National and destroyed by the PI and Ms. Briggs at the time of study close out.

Data Management
The data management team will be led by Dr. **Wang,** who will be responsible for supervising the interim and final analyses conducted by the Data Programmer and Data Analyst, Ms. Cheng will assume responsibility for all aspects of data management under the supervision of Adrienne Arietta and Dr. Wang, including assisting with data collection, data entry, data review, discrepancy checking and resolution, and quality assurance audits in accordance with FDA Good Clinical Practices guidelines (2007). Ms. Cheng will set up the database in RED Cap. A double-check system is used to assure accuracy and consistency of coding. Entered data are compared to the original data to assure accuracy. Weekly reports will be generated to show the status of all participants.

Data Quality Monitoring

The REDCap database from the FACE-TC pilot will be updated and expanded for this study, and the systems for data entry will be revised to address multi-site implementation. Data may be either directly entered through the website or will be captured on paper case report forms and the site RA will then enter this data into REDCap after the visit. The Data Manger will train RA-Assessors from each study site by visual teleconference to competency. To ensure accuracy, the data will go through some validity checks at the time of entry. The data manager will generate data queries based on range and logical checks from the database, prompted by the Data Center, as described below. This generates an email to RAs at the sites to resolve data issues. Routine edit checks will involve more complex queries. The intent in this approach is to allow sites to complete data entry promptly, and not slow them down except for very gross errors, and to query shortly thereafter should the data at the site show inconsistency. The Data Manager will monitor data to ensure timely and uniform collection.

Clinical and Translational Science Institute at Children's National (CTSI-CN)

In 2010, Children's National became the first pediatric institution to be funded by the prestigious NIH Clinical and Translational Science Award. We subsequently established the Clinical and Translational Science Institute at Children's National (CTSI-CN) with our partner institution, The George Washington University.

With the award, the NIH-funded General Clinical Research Center at Children's National was subsumed by the CTSI-CN's Participant and Clinical Interactions Resources (PCIR) component. Total space of the clinical research facility has grown from 3,205 sq ft in 2008 to 7,390 sq ft in 2010, a testament to the institution's commitment to clinical and community research. The unit currently includes:

2 patient rooms
2 patient interview rooms
3 patient treatment rooms
1 infusion room
2 specimen processing room
1 nursing station
1 patient/family waiting room
Rooms housing an array of testing equipment
1 conference room
Several staff offices

IRB
The CTSI-CN's Office for the Protection of Human Subjects recently developed an electronic portal (IRBear) where IRB applications and modifications are submitted for review, revision, approval, and renewal. The Principal Investigator and research team have access to all questions, documents, comments from the review board, and is a working space where all individuals involved with the study can access, revise, and route elements of the application.

Computers
The Principal Investigator has a Windows desktop computer with standard software, internet access through the hospital's intranet, and a networked all-in-one copier/printer/scanner/fax unit. IR Technical Services provides specialized computing support within the hospital with regard to computer hardware, operating systems, networking, Internet services, and desktop applications. Children's National deploys and maintains a variety of systems and services such as Unix, Novell, and Windows NT servers, file and print services, Email, and networked applications. The IR Operations team is responsible for 24 hour monitoring of the Wide-Area Network, and all server computer systems. Operations also perform system backups, report printing and distribution, and system failure resolution. The Application Development group provides user support for the hospital

operation management applications. Support includes table and file setup, process flow and assessment, design and development of programs and interfaces, report creation and generation, testing of software patches, enhancements and customizations, troubleshooting, and problem resolution to ensure that all applications are functioning properly.

Study Administrative Structure

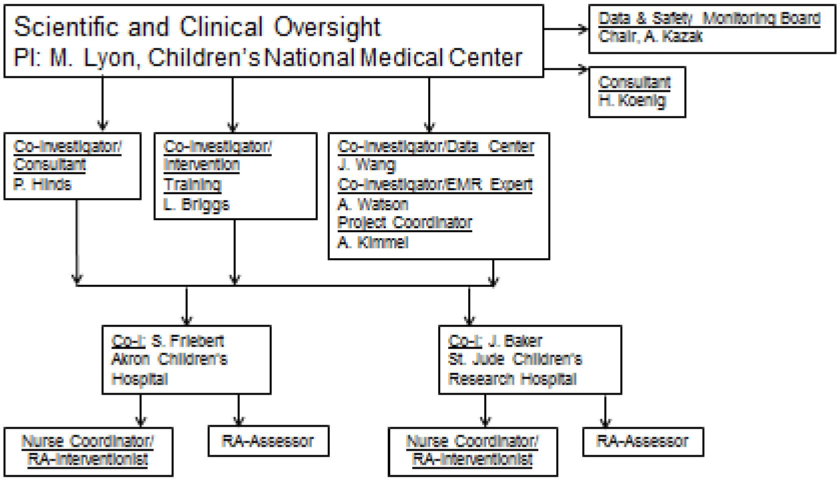

Akron Children's Hospital

Since its establishment in 1890, Akron Children's Hospital (ACH) has evolved into the largest pediatric health care system in northeast Ohio and the 8th largest children's hospital in the nation, operating two freestanding pediatric hospitals and offering services at 20 primary care and 67 pediatric specialty locations.

Our more than 30 pediatric specialties draw over 700,000 patients each year, and include children, teens and adults from all 50 states and around the world. ACH's main campus in downtown Akron houses regional centers for palliative care, genetics, fetal treatment, cancer and blood disorders, heart, orthopedics, pediatric trauma, pediatric intensive care, and level III neonatal intensive care, among others. As a teaching and research hospital, ACH is affiliated with Northeast Ohio Medical University (NEOMED), a community-based medical school which operates as an educational consortium with 18 community hospitals and three publicly-funded northeast Ohio universities (Kent State University, The University of Akron, and Youngstown State University). In addition to offering a wide range of specialized clinical care, ACH serves as an advocate for the health and welfare of children and a resource for parenting, community services, and consumer information.

Research

Clinical research is integral to the vision and mission of ACH and a long-standing pillar of the system's strategic plans. Our scope and reach attracts prospective research patients representing all socio-economic backgrounds including urban, suburban, and rural environments as well as patients from the Amish and Mennonite communities. ACH's Rebecca D. Considine Research Institute includes visionary centers of excellence dedicated to exploring forward-looking research domains with potential for novel investigative advances. With its core of dedicated research professionals and access to a diverse northeast Ohio and western Pennsylvania patient population, the institute is well-positioned to investigate the full gamut of illnesses and conditions afflicting children and burdening their families. The Institute is also home to several novel multidisciplinary endeavors not often found in hospital research centers.

The Rebecca D. Considine Research Institute is the central hub to provide clinical research resources for all ACH investigators. ACH's commitment and strong, persistent support for clinical research is reflected by a research endowment elevating the hospital's former Research Center to an institute. Naming it the Rebecca D. Considine Research Institute honors the wife of our sitting CEO, William Considine. Mr. Considine's one million dollar contribution to the institute's endowment further reflects major leadership support for pediatric research.

Numerous ACH investigators collaborate with our regional research partners at NEOMED, Kent State University, The University of Akron and Youngstown State University. The Research Institute has established multiple collaborative research teams with these regional academic institutions. To foster continued team development, ACH, with Kent State and NEOMED, provides pooled funding for competitively-awarded study teams to generate pilot data expected to culminate in major grant submissions within 2 years.

This momentum has allowed the Research Institute to build capacity and create specific research capabilities, such as:

- We provide an integrated research support system for ACH's medical staff engaged in the quest to improve patient care through science — including new treatments for rare or life-threatening childhood conditions.

- Our trained Clinical Services professionals provide parents significant opportunities to place their children in large-scale studies of new medicines and therapies, making such treatments available before widespread clinical acceptance.
- The Institute's accomplished research team pursues visionary, state-of-the-art advancements relevant to their academic interests, intellectual curiosity, knowledge of the field, and technical expertise.
- As a teaching hospital, we offer exceptional educational and research opportunities for fellows, students, and faculty—including mentoring hospital residents fulfilling research requirements.

Clinical research services, which are available to all ACH investigators through the Institute's professional staff provides support and direction with:

- study design
- protocol development
- budgeting and contracting
- regulatory and institutional review board compliance
- research database design and management
- biostatistical data analysis and interpretation
- medical communication and writing
- intellectual property protection
- technology transfer and commercialization

The Institute's clinical coordinator staff of nurses and medical assistants are fully trained and qualified to complete complex, large-scale clinical trials efficiently, effectively and safely. The success of the Institute's organizational structure is reflected in the hospital's institute-based Center for Nursing Research. This Center has achieved "exemplary" status for Magnet surveys. Likewise, ACH maintains long-standing successful collaborations with numerous multi-centered trial networks including the Cystic Fibrosis and the Pediatric Trials Network (PTN), a collaboration of the Eunice Kennedy Shriver National Institute of Child Health and Human Development and the Duke Clinical Research Institute, to study pediatric dosing and safety of prescription medications. Taken together, the Rebecca D. Considine Research Institute makes ACH a well-qualified, highly desirable site to conduct complex single and multi-site research initiatives by providing the expertise and capacity to successfully manage such clinical investigations.

Throughout the organization, numerous other research partnerships and national collaborations are also available clinical investigators and for patients and their families. A few of these include active participation in the Children's Oncology Group, Children's Hospital Association Hematology/Oncology and Pediatric Intensive Care collaboratives, leadership of and participation in a statewide initiative involving solutions for patient safety, the Vermont Oxford Network for neonatal intensive care, and V-PICU, an international database and research network for pediatric intensive care.

Clinical Care

The Haslinger Family Pediatric Palliative Care Division is housed on the 6th floor of the Considine Professional Building, located across the street from and connected by walkway to the main Akron campus hospital. The Palliative Care Center is staffed with the following:

- 2.85 FTE Palliative Care physicians, board certified in Hospice and Palliative Medicine
- 1 Pediatric Nurse Practitioner (2 FTE NP positions; one currently unfilled)
- 1 full-time Research Assistant
- 4 full-time nurse case managers (1 is clinical nurse specialist/advance practice provider)
- 4 social workers
- 1 bereavement coordinator (LISW by training)
- 2 physician fellows

- 2 full-time office staff personnel (Director's Secretary and Office Coordinator)
- 3 Expressive Therapy staff (Music Therapist, Art Therapist, Office Coordinator)
- 1 psychologist
- 1 chaplain
- Part-time ancillary staff in massage therapy, occupational therapy, physical therapy, child life
- 1 practice manager
- 1 volunteer coordinator (volunteer position) and over 100 Center-specific volunteers

The Center occupies 2330 square feet of space, with additional exam room/clinic space located next door to the Center. Contained in this space are 4 physician/NP offices, 1 separate office for the fellows, and office space for the secretarial, research, psychosocial and bereavement staff. A separate conference room houses the interdisciplinary team meetings and professional meetings, such as journal club, as well as offering a private space for family conferences. In the next door offices, the exam room is fully stocked with usual supplies; a second conference area is also available for private meetings with families as well as for simulation training with residents and fellows. All staff members have personal laptop or desktop computer devices and access to the electronic health record at work and remotely from off-site. Additionally, a 3000-foot Expressive Therapy Center, affiliated with the Palliative Care Center, is located centrally in the main hospital, offering art, music and any type of creative therapy imaginable to palliative care, oncology and other families served by Akron Children's Hospital.

The Palliative Care Center collaborates closely with the Showers Family Center for Childhood Cancer and Blood Disorders, located on the 5th floor of the main campus hospital building. The Showers Center comprises a fully-staffed outpatient clinic, including 8 treatment rooms, 4 separate exam rooms, 3 infusion bays, a physician work area, a large waiting area with specialized play area for patients and families, triage area, lab/phlebotomy space, and a chemo-pharmacy. The outpatient area is 7425 square feet, including nearby office space for physicians and Center support staff. The Center's clinic is located down the hall from the inpatient Hematology/Oncology hospital floor, which contains a 15-bed hepafiltered open unit, along with an additional 5 beds for hematopoietic stem cell transplant patients. The unit contains 2 kitchens (1 is separate for transplant patients), 2 play rooms for younger children and teens, a separate procedure room, and nursing and physician/team member work spaces and offices. The Showers Center maintains a separate clinic at Akron Children's Hospital's Boardman campus in nearby Youngstown, Ohio, where children and young adults residing in Ohio's Mahoning Valley (Trumbull, Columbiana and Mahoning Counties) can get the full-scope of cancer care closer to home. Clinic space there is available 4 days per week for outpatient evaluation and treatment of oncologic conditions; hospitalization is available on-site there for straightforward conditions, and transfer to main campus is arranged for children with more complicated medical issues or need for ICU-level care. Care is provided by 9.15 FTE physicians, as well as nurse practitioners, physician assistants, certified pediatric oncology nurses, on-site pharmacist and pharmD, social workers, clinical research associates, child life specialists, counselors and educators.

The Showers Center is a full COG member institution, ranks in the top 1/3 of pediatric cancer centers in the US in terms of numbers of new cancer cases treated each year, offers fellowship training in pediatric hematology-oncology, and is accredited by the American College of Surgeons Commission on Cancer. A separate late-effects cancer program and brain tumor/neuro-oncology program complement a full spectrum of care across diagnoses and ages.

Participant interviews at the Akron Children's site will be conducted in the Palliative Care exam room, conference room or private physician offices. Those conducted for hospitalized patients or patients being seen concurrently in the Showers Center will occur in an exam or treatment room, or in the Hematology/Oncology conference room co-located on the hallway between the inpatient unit and the outpatient clinic.

St. Jude Children's Research Hospital

Clinical Informatics provides application support for all Patient Care applications including implementation services, project management, upgrades, application support, decision support as well as traditional HIMS functions such as maintaining the accuracy and integrity of a hybrid medical record while insuring compliance with regulatory requirements, coding to support research and billing activities, release of information activities, and chart location to facilitate patient care and research. Individuals in these areas are the key points of contact for support in patient care departments for ongoing and new projects.

Enterprise Informatics includes the three key areas of Operations, Applications and Client Services. The Operations area includes foundational infrastructure including network, telecommunication, server, email, file and print services. Client Services provides support for desktop issues through the use of a centralized help desk and support technicians. The Application area provides support related to off-the-shelf system implementations, new web application and content development, and clinical integration for enterprise level administrative, clinical, and financial systems.

Research Informatics provides bioinformatics support, research computing infrastructure support including high-performance computing, software and database development for basic and clinical research, and the development of and updates to the Shared Resource Management (SRM) system.

> **Biostatistics** exists to provide statistical support to investigators of the St. Jude Children's Research Hospital (St. Jude) Cancer Center for peer-reviewed, funded grants and statistical design for institutional clinical and pre-clinical studies, as well as for basic science projects. The primary objectives of the Biostatistics Shared Resource are to provide Cancer Center investigators access to uniformly high quality, innovative statistical science; a centralized randomization system; access to statistical software; technical support for a web-based distributed data management system; and advice on data management issues.

Grants and Contract Management Office Services:

- Assists investigators in their endeavors to obtain extramural funding for research and training from the National Institutes of Health (NIH) and other funding agencies.
- Ensures that grant applications and progress reports meet the funding agencies' guidelines and federal regulations.
- Handles pre-award and post-award administrative activities.

The **Central Protocol and Data Monitoring Office (CPDMO)** provides a number of centralized clinical protocol-related services. The primary objectives of the CPDMO Shared Resource are to support clinical investigators by providing assistance in developing high quality clinical protocols, by providing a centralized clinical trial registration system, by performing ongoing routine monitoring of St. Jude investigator initiated clinical trials, by participating in the design and development of case report forms and data entry screens, by providing administrative support for the Clinical Trials - Scientific Review Committee (CT-SRC) and by providing investigational pharmacy support for therapeutic standard order sets. Protocol development, review, and activation

- Centralized research participant enrollment
- Monitoring the conduct of research
- Investigational studies pharmacist services
- Clinical research support for regional affiliates
- Support for Collaborating Study Sites

- Clinical Research contract negotiation and disbursements
- Clinical Research Education
- Administrative support for the CPSRMC

The **Clinical Trials - Scientific Review Committee (CT-SRC)** Services include:

- Scientific review of clinical protocols, including scientific rationale, study design, and appropriateness of biostatistical endpoints
- Monitors the progress of all institutional protocols to ensure that they adhere to the initial study design, that planned stopping criteria are followed, and that any observed toxicities are consistent with those expected
- Suspends or closes protocols that fail to meet these requirements

The **Health Information Management Services (HIMS)** include:

- Handles the coding, collection, processing, analysis, release and storage and retrieval of patient information contained in the medical record
- Assures that all medical record documents are accurate, complete and secure

The **Biomedical Library** and its electronic resources are available to St. Jude faculty and staff 24 hours a day, 7 days a week. The library staff is available to answer questions in person, by phone or email Monday-Friday. The staff also assists with searches and provides specialized training/orientation for library resources upon request.

The **Scientific Editing Resources** include:

- Editing scientific documents such as manuscripts, grant applications, book chapters, clinical protocols, abstracts, and poster presentations
- Substantive editing, copyediting, and proofreading
- Presenting workshops on writing, publication strategy, and good grantsmanship
- Offering consultation on grammar, punctuation, style, publication strategy, preparation and revision of documents, responses to reviewers' comments, etc

Word Processing Services provides support for research departments by typing and editing any type of document including:

- Grant applications: Word Processing assists in formatting grants correctly and inserting figures, illustrations and other content. Grants have top priority. Scientific Editing provides editorial assistance and Grants & Contract Management Office assists with all other aspects of grants.
- Biographical Sketches: Biographical sketches are kept for all faculty and postdocs for inclusion in grant applications. If biographical sketches are needed for a grant, Word Processing Center provides them.
- Manuscripts/Book Chapters: Manuscripts can be reformatted for various journals. Word Processing also types publications and inserts figures and tables. Book Chapters will be formatted according to templates sent by the editors.
- Curriculum Vitae: All faculty and postdoctoral fellow CV's are updated in WPC at least every six months.
- Meeting/Seminar/Conference Transcripts
- Interview Transcripts
- PowerPoint Presentations: Word Processing assists with Powerpoint presentations or posters. Files can either be e-mailed to the presenter or to the Biomedical Communications Department for production of posters.
- Policy/Procedure Manuals
- Flow charts & Organizational charts
- Merge documents
