# Supplementary material for: Effect of the Family-Centered Advance Care Planning for Teens with Cancer Intervention on Sustainability of Congruence About End-of-Life Treatment Preferences: A Randomized Clinical Trial
Source: JAMA Netw Open. 2022 Jul 12;5(7):e2220696. doi: 10.1001/jamanetworkopen.2022.20696 (PMC9277499; doi:10.1001/jamanetworkopen.2022.20696)
Supplement: Supplement 2. — eAppendix 1. Additional Details for Latent Class Analysis for Hypotheses 1 eTable 1. FAmily CEntered (FACE) Advance Care Planning Intervention eAppendix 2. Descriptive Statistics of the Congruence Measure by Latent Classes eTable 2. Selected Results of Longitudinal Latent Class Analysis (LLCA) for Congruence Between Patient and Family Over Time Regarding Treatment Preference in Four Medical Situations (N=117 Dyads) eTable 3. Effect of Intervention on Latent Class Classification, Results of Logistic Regression Model Using 3-step Procedure for the 4-time Point Data (T1-T4) (N=117 Dyads) eTable 4. Intervention Effect on Odds of Having Completed Any Advance Directive Documented in the Electronic Health Record at Study Close-out: Results of Logistic Regression (N=117 Dyads) eTable 5. Intervention Effect on Odds of Having Completed ACP Goals of Care Discussion Documented in Electronic Health Record at Study Close Out: Results of Logistic Regression (N=117 Dyads) eTable 6. Documentation in the Electronic Health Record of Any Advance Directive or Advance Care Planning Goals of Care Discussion at Study Close Out by Gender and Race eTable 7. Health Care Utilization 30 Days and 7 Days Prior to Death from the Electronic Patient Health Record Form (N=7 Adolescents) [file jamanetwopen-e2220696-s002.pdf]

Supplemental Online Content

Needle JS, Friebert S, Thompkins JD, et al. Effect of the Family-Centered Advance Care Planning for Teens With Cancer intervention on sustainability of congruence about end-of-life treatment preferences: a randomized clinical trial. *JAMA Netw Open*. 2022;5(7):e2220696. doi:10.1001/jamanetworkopen.2022.20696

eAppendix 1. Additional Details for Latent Class Analysis for Hypotheses 1

eTable 1. FAmily CEntered (FACE) Advance Care Planning Intervention

eAppendix 2. Descriptive Statistics of the Congruence Measure by Latent Classes

eTable 2. Selected Results of Longitudinal Latent Class Analysis (LLCA) for Congruence Between Patient and Family Over Time Regarding Treatment Preference in Four Medical Situations (N=117 Dyads)

eTable 3. Effect of Intervention on Latent Class Classification, Results of Logistic Regression Model Using 3-step Procedure for the 4-time Point Data (T1-T4) (N=117 Dyads)

eTable 4. Intervention Effect on Odds of Having Completed Any Advance Directive Documented in the Electronic Health Record at Study Close-out: Results of Logistic Regression (N=117 Dyads)

eTable 5. Intervention Effect on Odds of Having Completed ACP Goals of Care Discussion Documented in Electronic Health Record at Study Close Out: Results of Logistic Regression (N=117 Dyads)

eTable 6. Documentation in the Electronic Health Record of Any Advance Directive or Advance Care Planning Goals of Care Discussion at Study Close Out by Gender and Race

eTable 7. Health Care Utilization 30 Days and 7 Days Prior to Death from the Electronic Patient Health Record Form (N=7 Adolescents)

This supplemental material has been provided by the authors to give readers additional information about their work.

© 2022 Needle JS et al. *JAMA Network Open.*

eAppendix 1. Additional Details for Latent Class Analysis for Hypotheses 1

To determine the optimal number of latent classes, models with different numbers of classes were compared. Information criterion indices, such as Akaike's Information Criterion (AIC), Schwarz's Bayesian Information Criterion (BIC), and the sample-size-adjusted BIC, as well as likelihood ratio tests, such as the Lo-Mendell-Rubin (LMR) test, the adjusted Lo-Mendell-Rubin (ALMR) test, and Bootstrapped Likelihood Ratio (BLRT), were used for model comparison. The entropy statistic was used to assess the quality of class classification. Latent classes were defined based on the pattern of congruence probabilities over time conditional on class membership.

Once the latent classes of growth trajectories of congruence over time were identified and defined, we tested the intervention effect on the latent class membership, controlling for socio-demographic/clinical measures (e.g., age, gender, race/ethnicity, parental education, and family income). In testing such an effect, the newly developed 3-step approach[1,2] was used for modeling, considering the measurement errors in the latent class membership estimate that are inevitable in mixture models including LLCA. Conceptually speaking, the first step of the 3-step approach estimates an unconditional LPA model without any covariate. Then, measurement errors in latent class membership estimate were calculated in Step 2 based on the results of Step 1. And finally, the measurement errors were incorporated in modeling in Step 3. The implementation of the three steps of modeling was conducted simultaneously in modeling. Robust model estimator (e.g., MLR) in conjunction of the full information maximum likelihood (FIML) was used for model estimation. Missing at random (MAR), instead of missing completely at random (MCAR), can be assumed in MLR. MAR is a plausible assumption that allows missingness to be dependent on observed measures (e.g., intervention assignment). To determine the optimal number of latent classes, models with different numbers of classes were compared. Information criterion indices, such as Akaike's Information Criterion (AIC), Schwarz's Bayesian Information Criterion (BIC), and the sample-size-adjusted BIC, as well as likelihood ratio tests, such as the Lo-Mendell-Rubin (LMR) test, the adjusted Lo-Mendell-Rubin (ALMR) test, and Bootstrapped Likelihood Ratio (BLRT), were used for model comparison. The entropy statistic was used to assess the quality of class classification.

© 2022 Needle JS et al. *JAMA Network Open.*

Latent classes were defined based on the pattern of congruence probabilities over time conditional on class membership.

Once the latent classes of growth trajectories of congruence over time were identified and defined, we tested the intervention effect on the latent class membership, controlling for socio-demographic/clinical measures (e.g., age, gender, race/ethnicity, parental education, and family income). In testing such an effect, the newly developed 3-step approach[38,39] was used for modeling, considering the measurement errors in the latent class membership estimate that are inevitable in mixture models including LLCA. Conceptually speaking, the first step of the 3-step approach estimates an unconditional LPA model without any covariate. Then, measurement errors in latent class membership estimate were calculated in Step 2 based on the results of Step 1. And finally, the measurement errors were incorporated in modeling in Step 3. The implementation of the three steps of modeling was conducted simultaneously in modeling. Robust model estimator (e.g., MLR) in conjunction of the full information maximum likelihood (FIML) was used for model estimation. Missing at random (MAR), instead of missing completely at random (MCAR), can be assumed in MLR. MAR is a plausible assumption that allows missingness to be dependent on observed measures (e.g., intervention assignment).

1. Asparouhov, T., & Muthén, B. Auxiliary Variables in Mixture Modeling: Three-Step Approaches Using Mplus. *Structural Equation Modeling: A Multidisciplinary Journal*. 2014 21:329-341.
2. Vermunt, J.K. Latent class modeling with covariates: two improved three-step approaches. *Political Analysis*. 2010;18:450–469.

© 2022 Needle JS et al. *JAMA Network Open.*

eTable 1. FAmily CEntered (FACE) Advance Care Planning Intervention

	Session 1	Session 2	Session 3
**Session Foundation**	*Lyon Advance Care Planning (ACP) Survey - Adolescent and Surrogate Versions©* to set stage for EOL conversation. 45 Minutes average.	*Next Steps: Advance Care Planning Respecting Choices Interview Interview®* (Briggs and Hammes, 2012-2013)	The *Five Wishes©* is a legal document that helps a person express how they want to be treated if they are seriously ill and unable to speak for him/herself. Unique among living will and health agent forms - it looks to all of a person's needs: medical, personal, emotional, spiritual.
**Session Goals**	1. To assess the adolescents' values, spiritual and other beliefs, and life experiences with illness and EOL care.<br>2. To assess when to initiate ACP planning.<br>3. To assess the family member's (surrogate decision maker's) understanding of the adolescent's goals, values, spiritual and other beliefs, and life experiences with illness and EOL care.	1. To facilitate conversations and shared decision- making between the adolescent and surrogate about palliative care, providing an opportunity to express fears, values, spiritual and other beliefs and goals with regard to death and dying<br>2. To prepare the guardian/surrogate to be able to fully represent the adolescent's wishes	1. Which person the teen wants to make health care decisions for him/her;<br>2. The kind of medical treatment the teen wants;<br>3. How comfortable the teen wants to be;<br>4. How the teen wants people to treat him/her.<br>5. What teen wants loved ones to know;<br>6. Any spiritual or religious concerns teen may have.
**Session Process**	1. Orient family to study and issues.<br>2. Adolescent is surveyed privately;<br>3. Surrogate is surveyed privately with regard to what they believe their adolescent prefers.	Stage 1 assesses teen's understanding of condition;<br>Stage 2 explores teen's philosophy about EOL decision-making;<br>Stage 3 reviews rationale for future decisions teen would want surrogate to act on;<br>Stage 4 uses Statement of Treatment Preferences to describe scenarios/choices;<br>Stage 5 summarizes need for future conversations.	For adolescents under the age of 18, the Five Wishes © must be signed by their legal guardian. Processes, such as labeling feelings and concerns, as well as finding solutions to any identified problem, are facilitated. Appropriate referrals are made. *These sessions may include other family members or loved ones.

Source: Research-Tested Intervention Programs (R-TIPS). Site is no longer active.
https://rtips.cancer.gov/rtips/programDetails.do?programId=17054015

© 2022 Needle JS et al. *JAMA Network Open.*

eAppendix 2. Descriptive Statistics of the Congruence Measure by Latent Classes.

As shown in the **eTable 2** below, two latent classes (High vs. Low congruence) were defined based on the congruence pattern over four time points, immediately post-intervention, at 3-, 6- and 12-months post-intervention. In Class 1, the probability of having Perfect Congruence (i.e., agreement between adolescent patient and family in all 4 medical situations) is about 0.20 or above at all the time points; the probability of having Good Congruence (i.e., agreement between adolescent patient and family in 2-3 medical situations) is over 0.50 and kept increasing over time, reaching 0.80 at T4; and the probability of having Poor Congruence (i.e., agreement between adolescent patient and family in no more than one medical situation) is low, only 0.15 at T1, and declined over time, ending up to 0 at T4. On the contrary, in Class 2, the probability of having Poor Congruence is high, ranging from 0.44 (T1) to 0.74 (T4); and the probability of having Good Congruence and particularly Perfect Congruence, is very low at each time point, and kept declining over time. We, therefore, define Class 1 as High Congruence Class, and Class 2 as Low Congruence Class.

The model fit statistics are not for a specific LCA model, but for model comparisons. For this 2-class model, the Entropy statistic for quality of classification in this study is Entropy = 0.734 which is adequate. (The usual cut off is 0.70).

© 2022 Needle JS et al. *JAMA Network Open.*

eTable 2. Selected Results of Longitudinal Latent Class Analysis (LLCA) for Congruence Between Patient and Family Over Time Regarding Treatment Preference in Four Medical Situations (N=117 Dyads)

		Unconditional Probability	
Class 1: High Congruence (N=69)		0.59	
Class 2: Low Congruence (N=47)		0.41	
		**Conditional Probability**	
Outcome by Time		Class 1: High Congruence	Class 2: Low Congruence
T1	Poor Agreement[a]	0.15	0.44
	Good Agreement[b]	0.52	0.47
	Perfect Agreement[c]	0.33	0.09
T2	Poor Agreement[a]	0.14	0.56
	Good Agreement[b]	0.67	0.42
	Perfect Agreement[c]	0.19	0.02
T3	Poor Agreement[a]	0.06	0.80
	Good Agreement[b]	0.72	0.20
	Perfect Agreement[c]	0.21	0.00
T4	Poor Agreement[a]	0.00	0.74
	Good Agreement[b]	0.80	0.26
	Perfect Agreement[c]	0.20	0.00

Notes.
[a]: Agreement between adolescent patient and family in no more than one medical situation.
[b]: Agreement between adolescent patient and family in 2-3medical situations.
[c]: Agreement between adolescent patient and family in all 4 medical situations.
T1: Immediately post-Session 2
T2: 3-months post-intervention.
T3: 6-months post-intervention.
T4: 12-months post-intervention.
Latent classification was assigned by using 12 months data.

© 2022 Needle JS et al. *JAMA Network Open.*

eTable 3. Effect of Intervention on Latent Class Classification, Results of Logistic Regression Model Using 3-step Procedure for the 4-time Point Data (T1-T4) (N=117 Dyads)

Variable	β Estimate (Standard Error)	Two-Tailed P-Value
FACE®-TC pediatric Advance Care Planning	1.17 (0.55)	0.03
Age	-	
Young (≥14 years, <18 years)	0.55 (0.54)	0.30
Adolescent Gender		
Male	-	
Female	0.62 (0.54)	0.26
Adolescent Race		
Other	-	
White	1.38 (0.67)	0.04
Family Education		
<High School	-	-
High School or Less	-0.27 (0.61)	0.66
College/University or Higher	0.54 (0.77)	0.48
Family Household Income		
>2016 Federal Poverty Level	-	-
≤2016 Federal Poverty Level	0.51 (0.71)	0.47

[a]The Low Congruence class is treated as the reference group.

© 2022 Needle JS et al. *JAMA Network Open.*

eTable 4. Intervention Effect on Odds of Having Completed Any Advance Directive Documented in the Electronic Health Record at Study Close-out: Results of Logistic Regression (N=117 Dyads)

Variable	Odds Ratio (95% CI)
Intervention	
Control	-
FACE	19.20 (6.84-53.88) *
Race	
Others	-
White	0.65 (0.19-2.29)
Family Income	
>2016 Federal Poverty Level	-
≤2016 Federal Poverty Level	1.63 (0.52-5.06)
Gender	
Male	-
Female	1.17 (0.43-3.17)
Age	
Age 18-20 years	-
Age 14-17 years	1.03 (0.39-2.69)
Parental Education	
<=High School	-
Some College+	0.73 (0.20-2.67)

Note.

-: Reference group.

*: Statistically significant at 0.05 level.

© 2022 Needle JS et al. *JAMA Network Open.*

eTable 5. Intervention Effect on Odds of Having Completed ACP Goals of Care Discussion Documented in Electronic Health Record at Study Close Out: Results of Logistic Regression (N=117 Dyads)

Variable	Odds Ratio (95% CI)
Intervention	
Control	-
FACE	1.27 (0.35-4.70)
Race	
Others	-
White	0.42 (0.11-1.53)
Family Income	
>2016 Federal Poverty Level	-
≤2016 Federal Poverty Level	2.32 (0.65-8.23)
Gender	
Male	-
Female	1.65 (0.45-6.10)
Age	
Age 18-20 years	-
Age 14-17 years	1.24 (0.36-4.32)
Parental Education	
<=High School	-
Some College+	3.05 (0.34-27.71)

Note.
-: Reference group.

© 2022 Needle JS et al. *JAMA Network Open.*

eTable 6. Documentation in the Electronic Health Record of Any Advance Directive or Advance Care Planning Goals of Care Discussion at Study Close Out by Gender and Race

	intervention							
	Control				FACE-TC			
	Non-white male	Non-white Female	white male	white Female	Non-white male	Non-white Female	white male	white Female
**Any Advance Directive in Chart**								
**No**	1 (2.4%)	4 (9.5%)	14 (33.3%)	15 (35.7%)	0 (0%)	2 (2.7%)	6 (8.1%)	7 (9.5%)
**Yes**	1 (2.4%)	0 (0%)	0 (0%)	7 (16.7%)	7 (9.5%)	9 (12.2%)	21 (28.4%)	22 (29.7%)
**ACP Goals of Care Discussion Prior to Study**								
**No**	1 (2.4%)	3 (7.3%)	14 (34.1%)	19 (46.3%)	6 (8.5%)	6 (8.5%)	24 (33.8%)	25 (35.2%)
**Yes**	1 (2.4%)	0 (0%)	0 (0%)	3 (7.3%)	1 (1.4%)	4 (5.6%)	2 (2.8%)	3 (4.2%)

© 2022 Needle JS et al. *JAMA Network Open.*

eTable 7. Health Care Utilization 30 Days and 7 Days Prior to Death from the Electronic Patient Health Record Form (N=7 Adolescents)

Intervention group	Control	Control	Control	Control[a]	FACE-TC[b]	FACE-TC	FACE-TC
Age at baseline	16 years	18 years	16 years	18 years	18 years	17 years	14 years
Gender	Female	Female	Male	Male	Female	Female	Female
Race	White	White	African American	White	White	White	Asia
Type of Cancer	Brain Tumor	Leukemia	Relapsed AML	Brain Tumor	Solid Tumors	Leukemia	Solid Tumors
Advance Directive in Chart?	Yes	Yes	Yes	No	Yes	Yes	Yes
Involvement of Palliative Care Team	Yes	Yes	Yes	Yes	Yes	No	Yes
Did the patient die before completing the protocol?	Yes	Yes	No	No	Yes	Yes	Yes
Dyadic congruence at last completion of SoTP[c]	Low Congruence Latent Class 2 Poor	High Congruence Latent Class 1 Good	Low Congruence Latent Class 2 Poor	Low Congruence Latent Class 2 Poor	Missing data[b]	High Congruence Latent Class 1 Good	High Congruence Latent Class 1 Good
Adolescent Preference SoTP[c] Situation 1 Long hospitalization and low chance of survival	Continue	Continue	Unsure	Unsure	Missing data[b]	Stop	Stop
Adolescent Preference SoTP[c] Situation 2 3 months to live and side effects are serious	Continue	Continue	Unsure	Continue	Missing data[b]	Stop	Stop
Adolescent Preference SoTP[c] Situation 3 Physical impairment and 24-hour nursing care	Continue	Stop	Unsure	Continue	Missing data[b]	Stop	Stop

© 2022 Needle JS et al. *JAMA Network Open.*

Adolescent Preference SoTP[c]<br>Situation 4<br>Cognitive impairment	Unsure	Continue	Unsure	Unsure	Missing data[b]	Stop	Stop

© 2022 Needle JS et al. *JAMA Network Open.*

24-hour nursing care							
Palliative Care Note type	Palliative care note	Palliative care note	Palliative care note	Progress note, Family meeting note, Palliative care note	Progress note	Palliative care note	Palliative care note
Participants	Primary attending	ICU MD, QOL, FNP, mother, stepmother, stepfather	QOL FNP, QOL MD	Primary attending Spiritual care, Pain team, Social work, Study surrogate	Primary attending, Palliative Care Physician	Primary attending, Study Participant	Primary attending, Clinical Nurse Specialist, Study Participant, Study Surrogate,
Cause of death	Disease progression	cerebral edema secondary to refractory hyperammon-emia -- death due to cancer treatment-related causes	Relapsed AML	Disease progression[d]	Disease progression	Disease progression	Disease progression
Death anticipated	Yes	Yes	Yes	Yes	Yes	Yes	Yes
Number of hospitalizations in last 30 days of life	2	2	1	1	0	1	0
Total days of hospitalizations in last 30 days of life	6	16	14	12	0	30	0
Number of ICU admissions in last 30 days of life	1	1	1	1	0	1	0
Total days of ICU in last 30 days of life	3	6	14	12	0	30	0

© 2022 Needle JS et al. *JAMA Network Open.*

Total ER visits in last 30 days of life	1	0	0	1	0	0	0
Which chemotherapy did the patients get in last 30 days of life?	None	Aggressive	None	Aggressive	Palliative	Aggressive	None
If the patient got chemotherapy in last 30 days of life, please provide details (The name of the chemo, the desired effects of the chemo, ...):	NoA	Cytarabine Erwina Asparaginase IT Methotrexate and Cyt	NA		Pazopanib was started 9/16/2016 and given through January, 2017. It was stopped due to signs of toxicity. Desired affects were to slow progression of tumor growth.	Ponatinib, Daunorubicin, Etoposide and Ara-C started 10/23/19 in preparation for BMT	NA
Interventions utilized in last 30 days of life Chest compression)	yes	No	No	No	No	No	No
Interventions utilized in last 30 days of life Invasive mechanical ventilation	Yes	Yes	No	No	No	Yes	Yes
Interventions utilized in last 30 days of life Hemodialysis	No	Yes	No	No	No	Yes	No

© 2022 Needle JS et al. *JAMA Network Open.*

Interventions utilized in last 30 days of life BiPap, CPAP, nasal trumpet	No	No	No	Yes	No	No	No
Interventions utilized in last 30 days of life High-Flow oxygen therapy	No	No	No	Yes	No	Yes	No
Interventions utilized in last 30 days of life Defibrillation/cardiovert-sion	Yes	No	No	No	No	No	No
Interventions utilized in last 30 days of life New central line placed	No	Yes	No	No	No	No	No
Interventions utilized in last 30 days of life New chest tube placed	No	No	No	No	No	Yes	No
Interventions utilized in last 30 days of life Other	No	No	Yes	No	Yes	No	No
If Other, please explain	NA	NA	Discharged to SJ housing facility to be outpatient for holiday.	NA	At home in hospice.	NA	NA
Number of hospitalizations in last 7 days of life	1	1	1	1	0	1	0
Total days of hospitalizations in last 7 days of life	3	7	0	12	0	7	0
Number of ICU admissions in last 7 days of life	1	1	0	1	0	1	0
Total days of ICU in last 7 days of life	3	7	0	12	0	7	0

© 2022 Needle JS et al. *JAMA Network Open.*

Total ER visits:	0	0	0	1	0	0	0
Which chemotherapy did the patients get in last 7 days of life?	None	None	None	Aggressive	None	None	None
Interventions utilized in last 7 days of life<br>Chest compressions	Yes	No	No<br>EMS called following seizure.<br>EMS told no chest compressions or intubation	No	No	No	No
Interventions utilized in last 7 days of life<br>Invasive mechanical ventilation	No	No	No	No	Yes	No	Yes
Interventions utilized in last 7 days of life<br>Hemodialysis	No	Yes	No	No	No	No	No
Interventions utilized in last 7 days of life<br>bilevel positive airway pressure (BiPap)	No	No	No	Yes	No	No	No
Interventions utilized in last 7 days of life<br>High flow oxygen therapy	No	No	No	Yes	No	No	No
Interventions utilized in last 7 days of life<br>Cardiovascular resuscitation medications	Yes	No	No	No	No	No	No

© 2022 Needle JS et al. *JAMA Network Open.*

Interventions utilized in last 7 days of life Defibrillation/cardioversion	Yes	No	No	No	No	No	No
Interventions utilized in last 7 days of life New central line placed	No	Yes	No	No	No	No	No
Interventions utilized in last 7 days of life Other	No	Yes	Yes	No	Yes	No	No
If Other, please explain	NA	CRRT - Continuous Renal Replacement Therapy	Did not receive anything invasive. Received comfort care and a POLST was put in place. Died as outpatient at St Jude housing	NA	Did not have anything invasive. Received pain medications . Died at home with hospice	NA	NA
Preferred place of death	Inpatient hospice setting	Unknown	Outpatient	Unknown	Home with hospice	Unknown	Home with hospice
Place of Death	Inpatient hospice setting	Hospital ICU	Hospital Housing	Hospital ICU	Home with hospice	Hospital ICU	Home with hospice
Hospice involvement at end of life	None	None	Palliative care	None	Yes	None	Yes
If yes, how many days in hospice before death?	NA	NA	4	NA	71	NA	Missing
Evidence of bereavement support for sibling	No	Yes	"Whole family provided support"	Yes	No	Yes	Missing

© 2022 Needle JS et al. *JAMA Network Open.*

Evidence of bereavement support for parents	Yes	Yes	Yes	Yes	Yes	Yes	Missing

Notes:

a. Unblinded chart abstraction.
b. Adolescent completed baseline and Session 1 of the intervention, then withdrew due to disease progression.
c. SoTP: Statement of Treatment Preferences, last form completed prior to death.
d. Adolescent admitted with acute respiratory failure requiring BIPAP. Unable to wean and worsening mental status. Family chose to transition to comfort care, BiPap removed.

© 2022 Needle JS et al. *JAMA Network Open.*
