# Supplementary material for: Effect of the Family-Centered Advance Care Planning for Teens with Cancer Intervention on Sustainability of Congruence About End-of-Life Treatment Preferences: A Randomized Clinical Trial
Source: JAMA Netw Open. 2022 Jul 12;5(7):e2220696. doi: 10.1001/jamanetworkopen.2022.20696 (PMC9277499; doi:10.1001/jamanetworkopen.2022.20696)
Supplement: Supplement 3. — Data Sharing Statement [file jamanetwopen-e2220696-s00.pdf]

Data Sharing Statement

Needle. Effect of the Family-Centered Advance Care Planning for Teens with Cancer Intervention on Sustainability of Congruence About End-of-Life Treatment Preferences. *JAMA Netw Open*. Published July 12, 2022. doi:10.1001/jamanetworkopen.2022.20696

Data
Data available: Yes
Data types: Deidentified participant data, Data dictionary
How to access data: Please contact Dr. Maureen Lyon at mlyon@childrensnational.org for access to deidentified participant data and the data dictionary. Data available with publication.
When available: With publication

Supporting Documents
Document types: None

Additional Information
Who can access the data: Data will be made available to researchers whos proposed use of the data has been approved.
Types of analyses: Data will be made available for any research purpose.
Mechanisms of data availability: Data will be made available with investigator support after PI approval of the proposal.
Any additional restrictions: No additional restrictions, except time constraints due to COVID pandemic.
